# The Importance of HDL-Cholesterol and Fat-Free Percentage as Protective Markers in Risk Factor Hierarchy for Patients with Metabolic Syndrome

**DOI:** 10.3390/metabo12121217

**Published:** 2022-12-04

**Authors:** Ivona Mitu, Cristina-Daniela Dimitriu, Cristina Preda, Ovidiu Mitu, Irina-Iuliana Costache, Radu-Ștefan Miftode, Manuela Ciocoiu

**Affiliations:** 1 Department of Morpho-Functional Sciences II, University of Medicine and Pharmacy “Grigore T. Popa”, 700115 Iasi, Romania; 2 Department of Endocrinology, University of Medicine and Pharmacy “Grigore T. Popa”, 700115 Iasi, Romania; 3 1st Medical Department, University of Medicine and Pharmacy “Grigore T. Popa”, 700115 Iasi, Romania

**Keywords:** metabolic syndrome, body composition, body fat mass, body fat-free mass, adiponectin, leptin, prevalence, cardiometabolic risk

## Abstract

This research focused on establishing a hierarchy concerning the influence of various biological markers and body composition parameters on preventing, diagnosing and managing Metabolic Syndrome (MetS). Our cross-sectional cohort study included 104 subjects without any atherosclerotic antecedent pathology, organized in two groups (with and without MetS). All participants underwent clinical and anthropometric measurements, DEXA investigation and blood tests for all MetS criteria, together with adiponectin, leptin, insulin, uric acid and CRP. Based on mathematical logic, we calculated a normalized sensitivity score to compare the predictive power of biomarkers and parameters associated with MetS, upon the prevalence of MetS. Patients with MetS report higher levels of uric acid (*p* = 0.02), CRP (*p* = 0.012) and lower levels of adiponectin (*p* = 0.025) than patients without MetS. The top three biological markers with the highest predictive power of the prevalence of the disease are HDL, insulin, and adiponectin:leptin ratio, and the top three body composition parameters are trunk fat-free percentage, waist-height ratio and trunk fat percentage. Their high sensitivity scores differentiate them from all the other markers analysed in the study. Our findings report relevant scores for estimating the importance of cardiometabolic risks in the prevalence of MetS. The high rank of protective markers, HDL and trunk fat-free percentage, suggest that positive effects have a stronger association with the prevalence of MetS, than negative ones do. Therefore, this risk stratification study provides important support for prevention and management programs regarding MetS.

## 1. Introduction

Metabolic syndrome (MetS) is considered a cluster of several cardiometabolic risk factors associated with disrupted metabolism. Obesity and being overweight are at the centre of this condition, with approximately 13% of the world’s adult population being obese in 2016 [1]. The processes of this emerging pandemic of public health significance are not completely understood yet, but among the proposed mechanisms underlying chronic inflammation, insulin resistance and neurohormonal activation are key players in the progression of MetS [2]. According to the “World Health Organization”, obesity has tripled between 1975 and 2016; therefore, there is a need for better strategies to prevent and manage this disease [1].

One approach is to investigate and establish an importance scale of specific biomarkers or parameters closely linked with MetS and their influence on the disease prevalence. Studies have shown that adipokines, neuropeptides, inflammatory cytokines or prothrombotic factors are involved in the pathogenesis of MetS [3]. Therefore, besides the criteria used in MetS definition, other factors should be studied for better management of MetS. At the same time, body composition defined as the distribution of body weight (body fat, body lean, body muscle) over the trunk, legs and arms is still not enough explored in the management of MetS. Since obesity plays a centre role in the development of associated diseases [4,5], the International Diabetes Federation (IDF) introduced elevated waist circumference as a mandatory criterion in establishing the diagnosis of MetS [6]. Unfortunately, this measurement, as well as BMI, does not distinguish between body fat and body lean masses. Recent studies report sarcopenia as an independent risk factor for MetS, suggesting that the loss of muscle mass that occurs in the natural aging process is also associated with cardiometabolic risks [7,8,9,10]. In addition, it is well documented that adipose tissue itself and the dysfunction of body fat, known as adiposopathy, are associated with the development of diseases such as diabetes mellitus and atherosclerosis [11]. Therefore, a more thorough analysis of body masses and biological markers could provide a new hierarchy of parameters that better describe the risk profile of a patient concerning MetS.

## 2. Materials and Methods

### 2.1. Study Population

The study cohort included 104 participants aged 35–75 years, present for the first time or for follow up in the Cardiology Clinic, “St. Spiridon” Hospital, Iasi, Romania. All subjects had no antecedent atherosclerotic pathology and had no known chronic disease or had not followed treatment in the last 6 months for any cardiovascular or metabolic disease. The 2-year study was approved by the University Ethics Committee (number 1/27.07.2020) and all participants agreed and signed an informed consent prior to entering the study. The cohort was divided into two groups: one with MetS and another one without MetS. Based on the National Cholesterol Education Program—Third Adult Treatment Panel (NCEP-ATP III), the MetS criteria included waist circumference > 88 cm (women)/102 cm (men), glucose ≥ 100 mg/dL, HDL < 40 mg/dL (men)/<50 mg/dL (women), TG ≥ 150 mg/dL, and SBP/DBP ≥ 130/85 mmHg [6]. The MetS was present in patients with at least 3 abnormal components.

### 2.2. Data Collection

#### 2.2.1. Clinical and Anthropometric Measurements

At least 30 min prior to investigations, patients had not engaged in physical activity or not consumed caffeine rich products. For all participants, the same specialized personnel measured two times their height (stadiometer), waist circumference (flexible tape), hip circumference (flexible tape), abdominal and tricipital skinfold (Holtain-type caliper). For tricipital skinfold, halfway between the acromion process and olecranon process was considered and, for abdominal skinfold, 5 cm lateral of the umbilicus [12]. Waist circumference was measured between the last rib and the iliac crest at its smallest perimeter and hip circumference at the greater trochanter level [13].

Blood pressure was registered twice with a validated automatic device and cuffs of 3 sizes, according to arm circumference, after a 15-min rest in the seated position.

#### 2.2.2. Biochemical Measurements

All blood samples were collected in a clot–activator tube, during morning time, after a 12 h overnight fast and transported to the laboratory within 2 h, with a special isothermal bag for biological samples. A special timeframe between collection and centrifugation of maximum 15 min was considered for insulin. All centrifugation (Hettich Centrifuge Rotina 380 R) was performed at 4 °C and 4500 RPM for 10 min, and the serum samples obtained were either stored in separate tubes at −20 °C (for adiponectin, leptin, insulin) or used on the spot for measuring MetS markers with the spectrophotometric method (Roche Cobas c8000): TG kit applied the phosphate oxidase method, glucose kit applied the glucose-6-phosphate dehydrogenase method, HDL-chol kit applied the elimination/peroxidase method and total cholesterol kit applied the cholesterol oxidase method. Spectrophotometry was also used to measure uric acid, with the uricase and peroxidase method. CRP was measured by turbidimetry, with anti-CRP antibodies, using a 570 nm wavelength.

Insulin levels were measured using an enzyme-labeled chemiluminescent immunometric assay kit, Immulite 2000 Insulin, provided by Siemens (catalog number L2KIN2). For adiponectin and leptin measurements, the method of enzyme-linked immuno-sorbent assay (ELISA) kits was used, supplied by Biovendor-Laboratorni medicina a.s., Brno, Czech Republic: Adiponectin Human ELISA Competitive kit, CE-IVD, limit of detection 26 ng/mL, catalog number RD195023100 and Leptin Human ELISA, CE-IVD, limit of detection 0.2 ng/mL, catalog number RD191001100.

To calculate the homeostasis model assessment for insulin resistance (HOMA-IR), we applied the following formula reported in the literature [14]:

HOMA−IR=[insulin(μU/mL)×glucose(mg/dL)]/405

#### 2.2.3. Body Composition Measurements

We performed for each patient whole body composition determination with dual-energy-X-ray absorptiometry (DEXA)—Hologic QDR Delphi A fan-beam densitometer (Hologic Inc., Marlborough, MA, USA). The data report the distribution of adipose and lean tissue per whole body and separately for the trunk area. There is great confusion in the scientific literature regarding the correct use of the terms lean, lean body mass and fat free mass. In this article, we will use the term fat-free mass, which includes body lean tissue and bone mineral content (BMC) [15]. Fat mass index (FMI) and fat-free mass index (FFMI) were calculated as body fat mass and body fat-free mass, respectively, divided by height squared. We used these parameters to see if maybe they describe a more accurate relationship with MetS than BMI.

### 2.3. Prioritizing Parameters in the Prevalence of Metabolic Syndrome

The goal of our study is to identify the top biomarkers that present the highest predictive power concerning the prevalence of MetS. Therefore, we included in our analysis biological and clinical markers relevant to MetS, as well as body composition parameters, since obesity is the main criteria in diagnosing this disease.

We presented in the Results section and in the supplementary material the distribution and prevalence of subjects with MetS in the total population, at specific intervals for all biomarkers included. For standardization, the interval for each biomarker was considered half of the standard deviation (½ σ) for that specific biomarker. The distribution charts reported the number of participants with MetS in the respective biomarker interval. In the prevalence chart, the percentage of patients with MetS in that interval (*y*-axis) intersected with the point that represented half the interval (*x*-axis). The result reported a roughly linear relationship between biomarkers and the prevalence of MetS, supporting the use of derivatives that measure the sensitivity to change of the function value with respect to a change in its input variable. Because different biomarkers have different measurement units and rapport different intervals, we used a normalized sensitivity score defined by Criminisi et al. [16]. This score uses the chain rule for derivation with the help of the z-score of a measurement (number of standard deviations from the mean), thus providing a score with no unit. Therefore, this standardization made the comparison of biomarkers with respect to one another possible. The formula used is:

$$\mathrm{Sensitivity}\;\mathrm{score}={\;\sigma }_{\mathrm{parameter}}\times {\mathrm{coefficient}}_{\mathrm{parameter}}$$

The coefficient of the parameter is the slope of the regression line

### 2.4. Statistical Analysis

There were no missing data; therefore, no data substitution algorithm was necessary. All variables were analyzed using Microsoft Excel version 16.64 (Microsoft Corporation, Redmond, WA, USA) and SPSS version 23.0 (IBM Corporation, Armonk, NY, USA).

A Shapiro–Wilk test was used to evaluate if the data were normally distributed. Continuous variables were reported as mean ± SD (standard deviation) for normally distributed data or median and IQR (interquartile range) for non-normally distributed data. Categorical variables were expressed as frequencies (percentages).

One-way ANOVA test was used to report *p*-values for normally distributed data between the 2 groups, while the Mann–Whitney U test reported *p*-values for non-normally distributed data between the 2 groups. If one group presented normal distribution and one group did not, we applied the Mann–Whitney U test. The homogeneity of variances was tested with Levene’s test and, depending on the results, we reported mean rank or median values in order to correctly interpret the data. Chi-Square test was used for categorical variables.

Linear regression generated the coefficient of the parameter used in the formula stated for the sensitivity score, and R^2^ values were used to evaluate the goodness of fit of the model. Results were considered statistically significant if *p* < 0.05.

## 3. Results

### 3.1. Baseline Characteristics of the Study Population

The main characteristics of the study population are presented in Table 1. Regarding the entire study population, the *p*-value was greater than 0.05 for the Shapiro–Wilk test, therefore assuming normality of distribution, for WHR, abdominal and tricipital skinfold, trunk fat and fat-free percentage, HDL, non-HDL, DBP and uric acid. For the group with MetS and the group without MetS, we observed for most parameters a normal distribution in one group and a non-normal distribution in the other group. Therefore, we report the data accordingly: mean ± SD for normally distributed and median (ICQ) for non-normally distributed.

Homogeneity of variance between the two groups was assessed for the majority of parameters, except total and trunk fat-free mass in kg, glucose, HOMA-IR, TG, and adiponectin. For these parameters, we report the mean rank from Mann–Whitney U test to assess the difference between the two groups, while, for the others, we report the median (Table 2). We observe a statistically higher median for WC, WHtR, trunk total, fat and fat-free mass in kg, insulin, SBP, DBP, and CRP in the group with MetS. A higher mean rank is also reported for total and trunk fat-free mass, glucose, HOMA-IR, and TG. The group with MetS is also characterized by a statistically lower mean rank for adiponectin (*p* = 0.025) and lower mean for HDL-Chol (*p* = 0.001).

When comparing the median for the percentage of body fat mass and fat-free mass in patients with MetS, the results were not significantly different from those in patients without MetS. At the same time, the mass of body fat tissue in kilograms taken separately was decreased in patients with MetS, while the mass of body fat-free tissue in kilograms was increased. Considering these changes in the masses analysed, we aimed to further investigate how this increase affects the prevalence of MetS.

### 3.2. Sensitivity Score

We designed prevalence and distribution charts for biomarkers related to MetS, from biological and clinical parameters to body composition parameters. The results from the linear regression model for each marker and the sensitivity score values are reported in Table 3. Adiponectin, tricipital skinfold, trunk fat-free mass in kilograms, SBP and DBP showed no contribution to the variation of the prevalence of MetS (R^2^ < 0.02, *p* > 0.65); therefore, these markers were not taken into consideration when calculating the sensitivity score.

**Figure 1:**
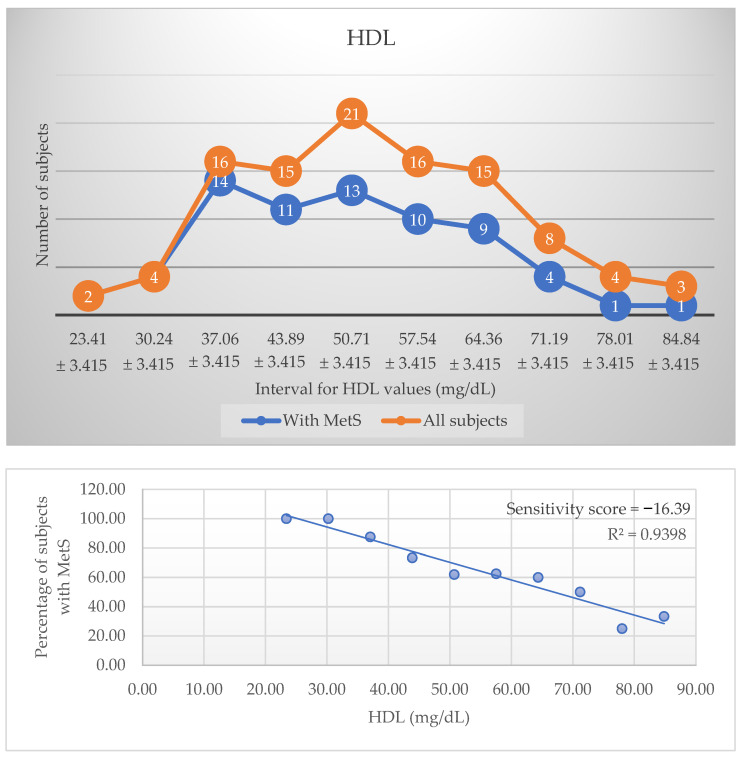
Distribution and prevalence charts for HDL.

**Figure 2:**
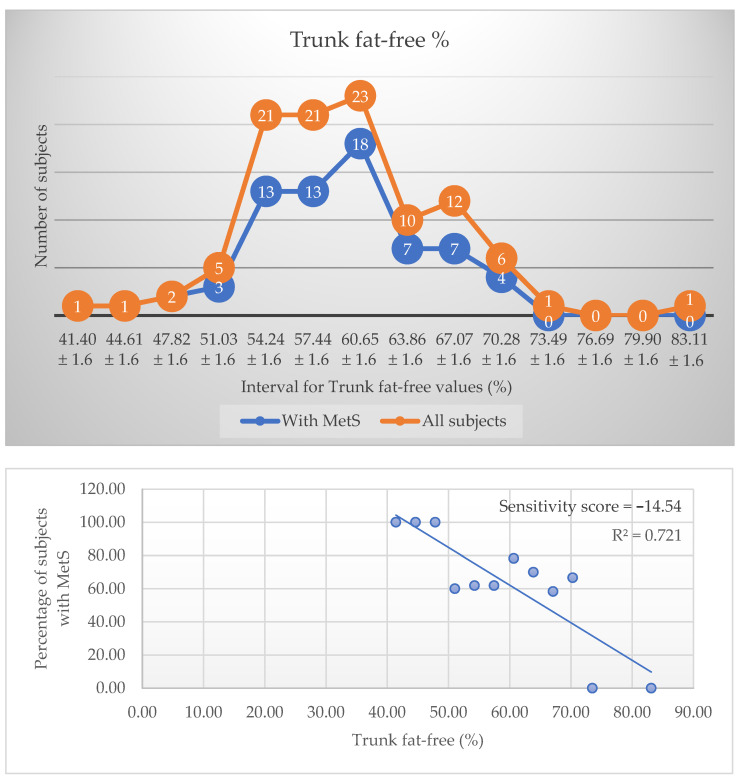
Distribution and prevalence charts for Trunk fat-free (%).

The prevalence and distribution charts for HDL and Trunk fat-free (%) as the parameters with the best R^2^ and sensitivity score values, each from their specific category, are presented in Figure 1 and Figure 2. The negative score indicates an inverse relationship with MetS, suggesting that higher values associate with a low prevalence of this disease. The top three biological markers are HDL, insulin, and ALR and the top three body composition parameters are trunk fat-free percentage, waist-height ratio (WHtR), and trunk fat percentage. Their high sensitivity score differentiates them from all the other parameters, and they are considered to have the most influence on the condition prevalence (Table 3). Figure 1 and Figure 2 were introduced for exemplification. All the other charts can be consulted in the Supplementary material (S1).

When we analyse more closely the distribution charts, we observe that, where both lines representing patients with MetS and without intersect, this means that all patients in that interval have the disease; therefore, the prevalence will be high.

## 4. Discussion

The main findings of our study address the sorting order of the most relevant parameters that influence MetS prevalence. From all body composition parameters measured by DEXA, only trunk fat-free and trunk fat percentages are statistically significant when considering MetS prevalence, with a high sensitivity score. Our results support other studies, suggesting that abdominal fat is the most important adipose tissue when discussing metabolic syndrome [17,18]. It is more relevant to calculate the trunk body masses of a patient than their total body masses. Even though there was no difference in the means of trunk fat percentages in subjects with and without MetS, in a more thorough analysis, we observed an increase in both masses separately. We underline the high importance of these parameters and therefore the necessity of pursuing their values in prevention and management of MetS. These are not only superior to BMI, WC, and HP in assessing the prevalence of MetS, but also more important than WHR and WHtR, which are considered solid prediction tools for cardiometabolic diseases [19,20]. A robust systematic review and meta-analysis on more than 300,000 subjects of various nationalities and ethnic groups showed that WHtR is a more reliable predictor than WC and BMI for obesity-related cardiometabolic diseases and therefore justifying its use in clinical practice [19]. Also in our study, WHtR and WHR are in the top body composition parameters that influence MetS prevalence, but the high sensitivity score comes with an important amendment. A small deviation from the real value of a parameter leads to an important change in the prevalence of the disease. Therefore, even though WHtR or any other anthropometric measure are easy to perform and require no cost, these measures are prone to human error and they should be reconsidered. In contrast, body masses are measured by high performing devices and therefore conclude to a standardized and objective value. Trunk fat-free percentage is considered the topmost important body composition parameter in our study. Trunk fat percentage has a lower sensitivity score than WHtR, but a higher R^2^ value, meaning that 72% of the variability observed in the prevalence of MetS is explained by trunk fat percentage, compared to only 62% for the WHtR. These are important arguments for considering mass percentages as a priority in the management of MetS.

Of all biological markers analysed, HDL was the most relevant one, with a very strong and negative coefficient of determination: 94% of the variability in the prevalence of the disease is explained by HDL values. This relevant relationship is observed in other studies as well, where low HDL is independently associated with incident MetS [21,22] and, moreover, the modified structure of HDL (sphingosine-1-phosphate depleted) in these patients leads to a decrease in the activation of endothelial nitric oxide synthase, therefore promoting atherosclerosis progression [23]. Another possible mechanism that mediates cardiovascular disease risk could be the change in cholesterol efflux capacity [24,25], possibly explained by low adiponectin and apoA-I levels [26].

In our study population, patients with MetS present a higher mean value of uric acid (*p* = 0.02), confirming data in the literature that indicate a linear dose–response relationship between this biomarker and the risk of MetS [27]. Experimental studies suggest that hyperuricemia may mediate insulin resistance by high levels of mitochondrial oxidative stress [28].

Results in our study show that insulin is more relevant than glucose for MetS prevalence, with a score value more than double when compared to glucose. Glucose was the last of the six statistically significant biological markers for the prevalence of MetS, suggesting a low influence on MetS prevalence. The NCEP-ATP III discarded insulin as a criterion in diagnosing MetS because measurements are laborious and not well standardized [6]. On the contrary, WHO and the European Group for the Study of Insulin Resistance (EGIR) continue to include insulin resistance in their definition of MetS [6,29]. The results of our study reiterate the importance insulin has on different stages of MetS and on its prevention, supporting the continuous research for a gold standard technique that will also provide the advantage of direct comparisons between studies [30].

Together with HDL and insulin, ALR is an important marker that associates well with the prevalence of MetS. Its high values suggest protective anti-inflammatory, antiatherogenic properties, which are known to be positively associated with high HDL values [31]. Taken separately, adiponectin mean is statistically lower in patients with MetS than in patients without MetS, while leptin values do not differ significantly between the two groups. The adipokine profile for this population and correlations with the other biomarkers have been already discussed in previous research [32]. ALR was already proposed as a valid biomarker of dysfunctional adipose tissue [33]. Our research highlights the fact that this parameter is in the top three markers that best associate with MetS prevalence, more important than TG, HOMA-IR, or glucose.

There are several strengths concerning our study. The first one is that we demonstrated the close relationship between the prevalence of MetS and body masses, as opposed to comparing it to classical parameters. The prevalence of cardiometabolic disorders significantly decreases once patients build up more abdominal fat-free mass and increases together with abdominal fat. The second strength of our study is that we stratified MetS risks with the help of biological and clinical markers. The third strength is the criteria used for selecting our study cohort, respectively no antecedent atherosclerotic pathology and no known chronic disease or no treatment in the last 6 months for any cardiometabolic disease. Thus, the bias of other diseases or medication has been reduced. In addition, subjects that enrolled in our study had not been given a diagnosis of MetS beforehand, making our population even more representative for advocating the early assessment of this complex pathology.

On the other hand, our study has some limitations: (a) comparison across genders was not feasible (subjects were mostly women), (b) the regression line between prevalence and biomarkers would ideally need more subjects to prove its linearity, (c) the biomarker intervals with no patients included were disregarded in order not to mistakenly distort the regression. We need to investigate into a more consistent population for other types of regression such as exponential, logarithmic or polynomial in order to validate or invalidate the best model of linear regression for each selected parameter. Nevertheless, low *p*-values and high R^2^ values justify our approach for the most relevant parameters concerning MetS.

Our findings show two documented and relevant possibilities of estimating with a high precision the risk exposure of the patient towards developing MetS: measuring HDL, insulin or ALR in the laboratory or determining trunk fat-free mass, WHtR or trunk fat mass. The choice remains to both medical personnel and patients and depends on the given possibilities.

## 5. Conclusions

Based on the impact parameters manifesting towards MetS prevalence, our study established a hierarchical order of those highly associated with MetS. The protective markers, HDL and trunk fat-free percentage report having the strongest association with MetS, each in their own category, suggesting that higher values associate with a low prevalence. Trunk fat-free percentage is more important than body fat mass percentage and HDL is more important than ALR or TG. This stratification provides important support for prevention and management programs of this condition. Therefore, we suggest, as a key direction in strategies for MetS, maintaining functional and high HDL, along with a strong representation of body fat-free mass in patients.

## Figures and Tables

**Table 1:** Baseline characteristics of the study population.

	All Subjects (*n* = 104)	Patients without MetS (*n* = 35)	Patients with MetS (*n* = 69)	*p* *
	Mean (Lower Bound-Upper Bound) ± SD OR Median (IQR)	Mean (Lower Bound-Upper Bound) ± SD OR Median (IQR)	Mean (Lower Bound-Upper Bound) ± SD OR Median (IQR)	
**Demographic and anthropometric parameters**				
Age	62 (12)	58.29 (55.7–60.88) ± 7.54	63 (13)	
Gender **				
Female	74.04% (77)	82.86% (29)	69.56% (48)	
Male	25.96% (27)	17.14% (6)	30.44% (21)	0.16
BMI (kg/m^2^)	30.99 (5.83)	30.61 (28.87–32.36) ± 5.08	31.01 (6.10)	
WC (cm)	106 (16)	100 (49)	110 (18)	
HC (cm)	113 (12.8)	111.86 (108.65–115.06) ± 9.33	115 (13)	
WHtR	0.64 (0.10)	0.62 (0.60–0.65) ± 0.07	0.65 (0.08)	
WHR	0.94 (0.93–0.95) ± 0.06	0.92 (0.90–0.94) ± 0.06	0.96 (0.94–0.97) ± 0.06	*0.003*
Abdominal skinfold (mm)	36.22 (34.63–37.81) ± 8.17	35.66 (32.6–38.71) ± 8.90	36.51 (34.63–38.39) ± 7.82	0.62
Tricipital skinfold (mm)	27.06 (25.61–28.50) ± 7.43	27 (24.41–29.59) ± 56.65	27.09 (25.30–28.88) ± 7.44	0.95
**Body composition parameters assessed by DEXA**				
Total Fat (kg)	34.16 (9.22)	33.56 (30.34–36.78) ± 9.37	34.03 (9.26)	
Total fat-free (kg)	49.07 (15.07)	47.79 (9.52)	51.44 (20.40)	
Trunk fat (kg)	17.32 (5.76)	16.25 (14.23–18.27) ± 5.88	18.40 (6.14)	
Trunk fat-free (kg)	24.96 (8.65)	23.78 (3.99)	27.17 (25.75–28.60) ± 5.93	
Trunk total (kg)	42.40 (13.73)	39.13 (10.28)	45.50 (13.76)	
Total fat (%)	41.40 (8)	42.30 (8.30)	40.07 (38.55–41.59) ± 6.32	
Total fat-free (%)	58.90 (7.95)	57.70 (8.30)	60.12 (58.59–61.64) ± 6.34	
Trunk fat (%)	40.30 (39.05–41.55) ± 6.42	41.30 (9.90)	40.82 (39.34–42.29) ± 6.13	
Trunk fat-free (%)	59.70 (58.45–60.95) ± 6.42	58.70 (9.90)	59.93 (58.41–61.44) ± 6.32	
FMI (kg/m^2^)	12.59 (4)	12.45 (11.31–13.58) ± 3.31	12.60 (3.86)	
FFMI (kg/m^2^)	18.28 (4.30)	18.17 (17.24–19.09) ± 2.69	19.28 (18.57–19.99) ± 2.96	0.06
**MetS associated biomarkers**				
Glucose (mg/dL)	103 (22)	94 (10)	105 (27.50)	
Insulin (μUI/mL)	15 (12.07)	9.69 (10.24)	16.40 (17.95)	
HOMA-IR	3.78 (3.60)	2.19 (2.68)	4.48 (5.70)	
TG (mg/dL)	135 (83.5)	97.63 (88.10–107.15) ± 27.73	168 (104)	
HDL-Chol (mg/dL)	52.63 (49.98–55.29) ± 13.65	59.03 (54.81–63.25) ± 12.29	49.39 (46.21–52.57) ± 13.23	*0.001*
Non-HDL (mg/dL)	155.97 (147.24–164.70) ± 44.90	153.91 (140.09–167.73) ± 40.23	157.01 (145.64–168.39) ± 47.34	0.74
SBP (mmHg)	135 (21.8)	125 (21)	139 (18.50)	
DBP (mmHg)	85.94 (83.61–88.28) ± 12.01	79 (12)	90 (14.5)	
Leptin (ng/dL)	19.63 (28.45)	19.87 (30.40)	19.40 (28.50)	
Adiponectin (μg/dL)	13.02 (6.45)	15.74 (13.75–17.73) ± 5.80	12.84 (3.69)	
ALR	1.98 (3.43)	1.75 (1.94)	2.12 (3.45)	
Uric acid (mg/dL)	5.52 (5.21–5.83) ± 1.60	5.01 (4.63–5.39) ± 1.10	5.77 (5.35–6.19) ± 1.75	*0.02*
CRP (mg/dL)	0.32 (0.38)	0.20 (0.35)	0.40 (0.61)	

* *p*-values only for parameters that have normal distribution in both groups (based on one-way ANOVA). ** values are reported under the form of percentage (number of subjects). Note: SD = standard deviation, IQR = interquartile range, WC = waist circumference, HC = hip circumference, WHtR = waist–height ratio, WHR = waist–hip ratio, Total fat(%) = total fat mass percentage, Total fat-free (%) = total fat-free mass percentage, Trunk fat (%) = trunk fat mass percentage, Trunk fat-free (%) = trunk fat-free mass percentage, FMI = fat mass index, FFMI = fat free mass index, SBP = systolic blood pressure, DBP = diastolic blood pressure, ALR = adiponectin–leptin ratio.

**Table 2:** Comparison of parameters between the group with MetS and the group without MetS.

	Patients without MetS (*n* = 35)	Patients with MetS (*n* = 69)	*p*
	Median OR Mean Rank (*)	Median OR Mean Rank (*)	
Age	61	63	0.202
BMI (kg/m^2^)	31	31.01	0.133
WC (cm)	100	110	*0.001*
HC (cm)	112	115	0.120
WHtR	0.62	0.65	*0.015*
Total Fat (kg)	34.32	34.03	0.431
Total fat-free (kg)	43.89 *	56.87 *	*0.038*
Trunk fat (kg)	16.52	18.40	*0.025*
Trunk fat-free (kg)	42.14 *	57.75 *	*0.013*
Trunk total (kg)	39.13	45.50	*0.03*
Total fat (%)	42.30	40.80	0.573
Total fat-free (%)	57.70	59.20	0.573
Trunk fat (%)	41.30	40.50	0.522
Trunk fat-free (%)	58.70	59.50	0.522
FMI (kg/m^2^)	12.59	12.60	0.888
Glucose (mg/dL)	31.29 *	63.26 *	*<0.001*
Insulin (μUI/mL)	9.69	16.40	*<0.001*
HOMA-IR	33.80 *	61.99 *	*<0.001*
TG (mg/dL)	27.59 *	65.14 *	*<0.001*
SBP (mmHg)	125	139	*<0.001*
DBP (mmHg)	79 (12)	90 (14.5)	*0.001*
Leptin (ng/dL)	19.87	19.40	0.804
Adiponectin (μg/dL)	61.80 *	47.78 *	*0.025*
ALR	1.75	2.12	0.264
CRP (mg/dL)	0.20	0.40	*0.012*

* mean rank is reported. Note: WC = waist circumference, HC = hip circumference, WHtR = waist-height ratio, Total fat (%) = total fat mass percentage, Total fat-free (%) = total fat-free mass percentage, Trunk fat (%) = trunk fat mass percentage, Trunk fat-free (%) = trunk fat-free mass percentage, FMI = fat mass index, SBP = systolic blood pressure, DBP = diastolic blood pressure, ALR = adiponectin–leptin ratio.

**Table 3:** Hierarchical order of biomarkers relevant for MetS prevalence.

Rank	Biomarker	Score	Coefficients		R	R^2^	*p*
			Intercept	Variable			
*Biological and clinical parameters*							
1	HDL-chol (mg/dL)	−16.39	130.33	−1.20	0.97	0.94	*<0.001*
2	Insulin (μUI/mL)	16.36	36.37	1.43	0.86	0.73	*0.003*
3	ALR	−13.44	84.42	−15.60	0.87	0.76	*0.002*
4	TG (mg/dL)	11.88	47.28	0.14	0.74	0.55	*0.01*
5	HOMA-IR	9.83	58.60	2.14	0.78	0.61	*0.01*
6	Glucose (mg/dL)	7.17	49.74	0.20	0.66	0.43	*0.04*
7	CRP (mg/dL)	6.81	61.17	13.22	0.90	0.80	0.10
8	Uric acid (mg/dL)	5.57	57.97	3.47	0.40	0.16	0.19
9	Leptin (ng/dL)	5.16	59.47	0.35	0.49	0.24	0.21
10	nonHDL (mg/dL)	4.74	51.64	0.10	0.39	0.15	0.26
*Body composition parameters*							
1	Trunk fat-free (%)	−14.54	198.16	−2.27	0.85	0.72	*<0.001*
2	WHtR	13.81	−74.39	202.34	0.79	0.62	*0.004*
3	Trunk fat (%)	11.81	−10.16	1.84	0.85	0.72	*<0.001*
4	WHR	10.06	−88.25	162.03	0.76	0.57	*0.01*
5	Total fat (%)	9.74	7.65	1.53	0.54	0.29	0.11
6	Total fat-free (%)	−9.33	156.32	−1.47	0.51	0.26	0.13
7	FMI (kg/m^2^)	8.91	23.64	2.45	0.55	0.31	0.06
8	HC (cm)	8.83	−38.36	0.84	0.51	0.26	0.11
9	Total fat (kg)	8.75	23.03	0.83	0.50	0.25	0.11
10	WC (cm)	8.46	−9.20	0.68	0.49	0.24	0.17
11	Abdominal skinfold (mm)	8.05	25.83	0.98	0.44	0.19	0.16
12	Trunk fat (kg)	7.98	28.27	1.18	0.51	0.26	0.13
13	Total fat-free (kg)	7.77	28.90	0.68	0.41	0.17	0.27
14	FFMI (kg/m^2^)	7.12	21.26	2.45	0.56	0.31	0.12
15	BMI (kg/m^2^)	7.09	17.60	1.32	0.43	0.18	0.19
16	Tricipital skinfold (mm)	6.96	43.98	0.94	0.58	0.34	0.08

Note: the parameters are listed in each category in a descendent order from the highest sensitivity score to the lowest; ALR = adiponectin-leptin ratio, Trunk fat-free (%) = trunk fat-free mass percentage, WHtR = waist-height ratio, Trunk fat (%) = trunk fat mass percentage, WHR = waist-hip ratio, Total fat (%) = total fat mass percentage, Total fat-free (%) = total fat-free mass percentage, FMI = fat mass index, HC = hip circumference, WC = waist circumference, FFMI = fat free mass index.

## Data Availability

Data supporting reported results are available from the corresponding authors. Data is not publicly available due to privacy.

## Supplementary Materials

The following supporting information can be downloaded at: https://www.mdpi.com/article/10.3390/metabo12121217/s1, Figure S1: Distribution and prevalence charts for Insulin, Figure S2: Distribution and prevalence charts for ALR (adiponectin:leptin ratio), Figure S3: Distribution and prevalence charts for Triglycerides, Figure S4: Distribution and prevalence charts for HOMA-IR, Figure S5: Distribution and prevalence charts for Glucose, Figure S6: Distribution and prevalence charts for CRP, Figure S7: Distribution and prevalence charts for Uric acid, Figure S8: Distribution and prevalence charts for Leptin, Figure S9: Distribution and prevalence charts for non-HDL, Figure S10: Distribution and prevalence charts for WHtR (waist-height ratio), Figure S11: Distribution and prevalence charts for Trunk fat (%), Figure S12: Distribution and prevalence charts for WHR (waist-hip ratio), Figure S13: Distribution and prevalence charts for Total fat (%), Figure S14: Distribution and prevalence charts for Total fat-free (%), Figure S15: Distribution and prevalence charts for FMI (fat mass index), Figure S16: Distribution and prevalence charts for HC (hip circumference), Figure S17: Distribution and prevalence charts for Total fat (kg), Figure S18: Distribution and prevalence charts for WC (waist circumference), Figure S19: Distribution and prevalence charts for Abdominal skinfold, Figure S20: Distribution and prevalence charts for Trunk fat (kg), Figure S21: Distribution and prevalence charts for Total fat-free (kg), Figure S22: Distribution and prevalence charts for FFMI (fat free mass index), Figure S23: Distribution and prevalence charts for BMI, Figure S24: Distribution and prevalence charts for Tricipital skinfold.
