# Supplementary material for: The Importance of HDL-Cholesterol and Fat-Free Percentage as Protective Markers in Risk Factor Hierarchy for Patients with Metabolic Syndrome
Source: Metabolites. 2022 Dec 4;12(12):1217. doi: 10.3390/metabo12121217 (PMC9784319; doi:10.3390/metabo12121217)
Supplement: Supplementary file 1 [file metabolites-12-01217-s001.zip › metabolites-1988866-supplementary.pdf]

Distribution and prevalence charts for biological markers in descending order of their influence towards MetS

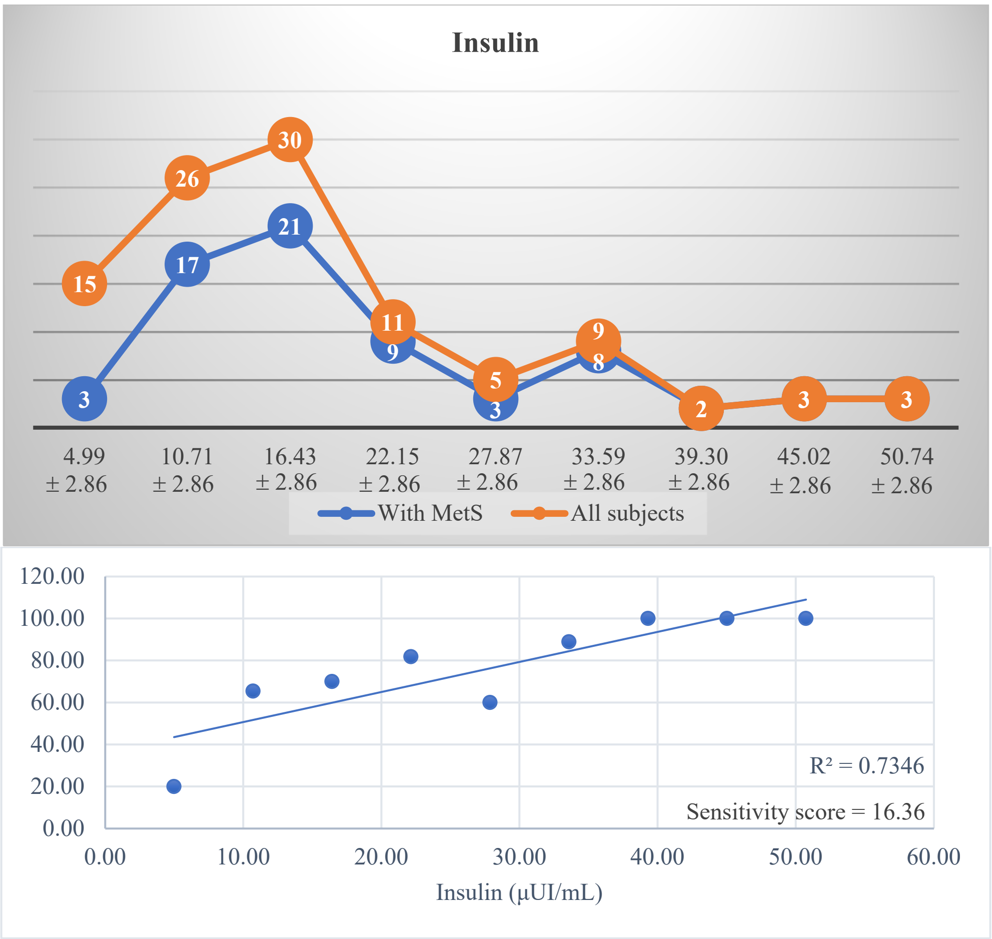

Figure S1. Distribution and prevalence charts for Insulin

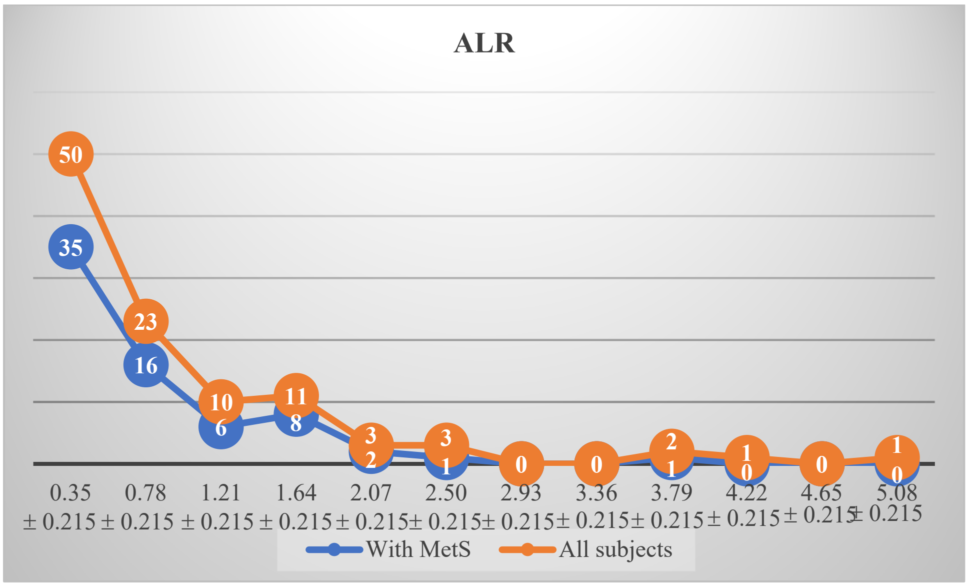

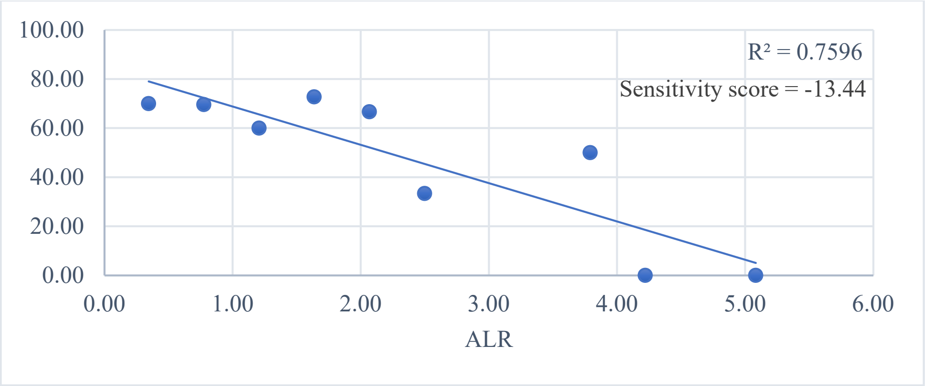

Figure S2. Distribution and prevalence charts for ALR (adiponectin:leptin ratio)

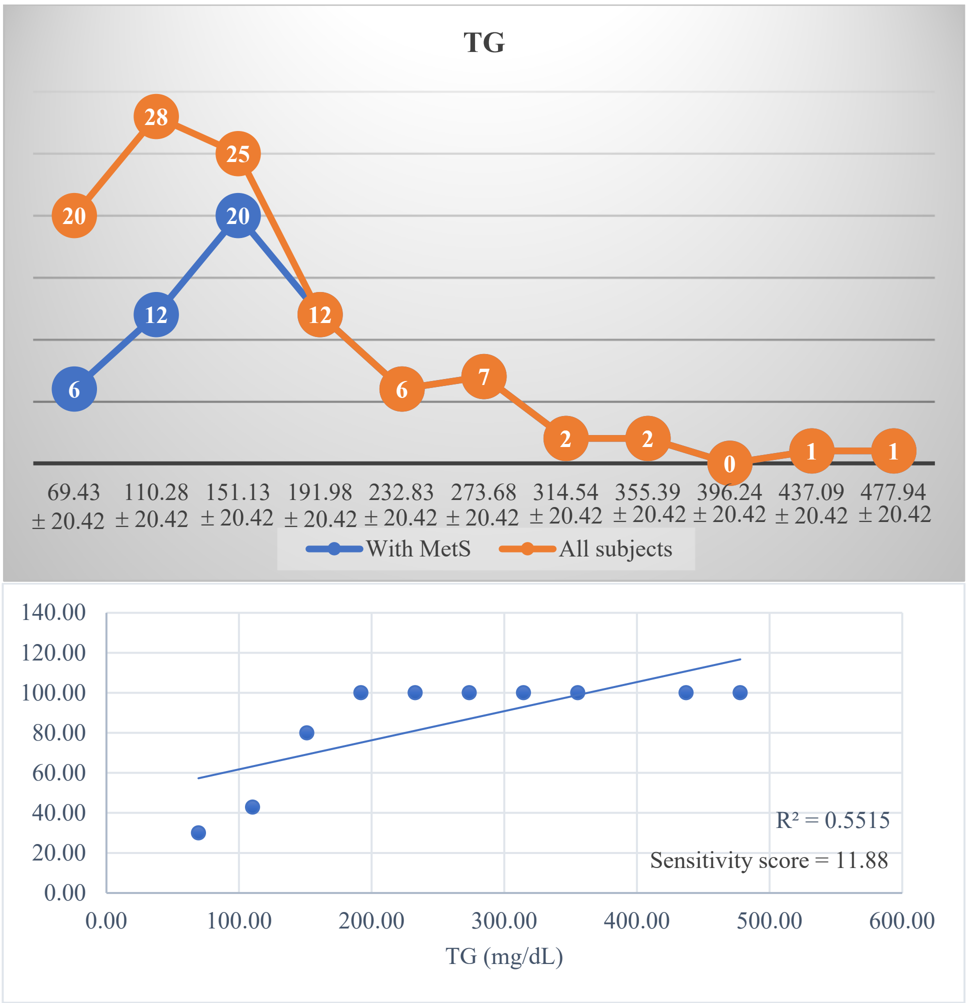

Figure S3. Distribution and prevalence charts for Triglycerides

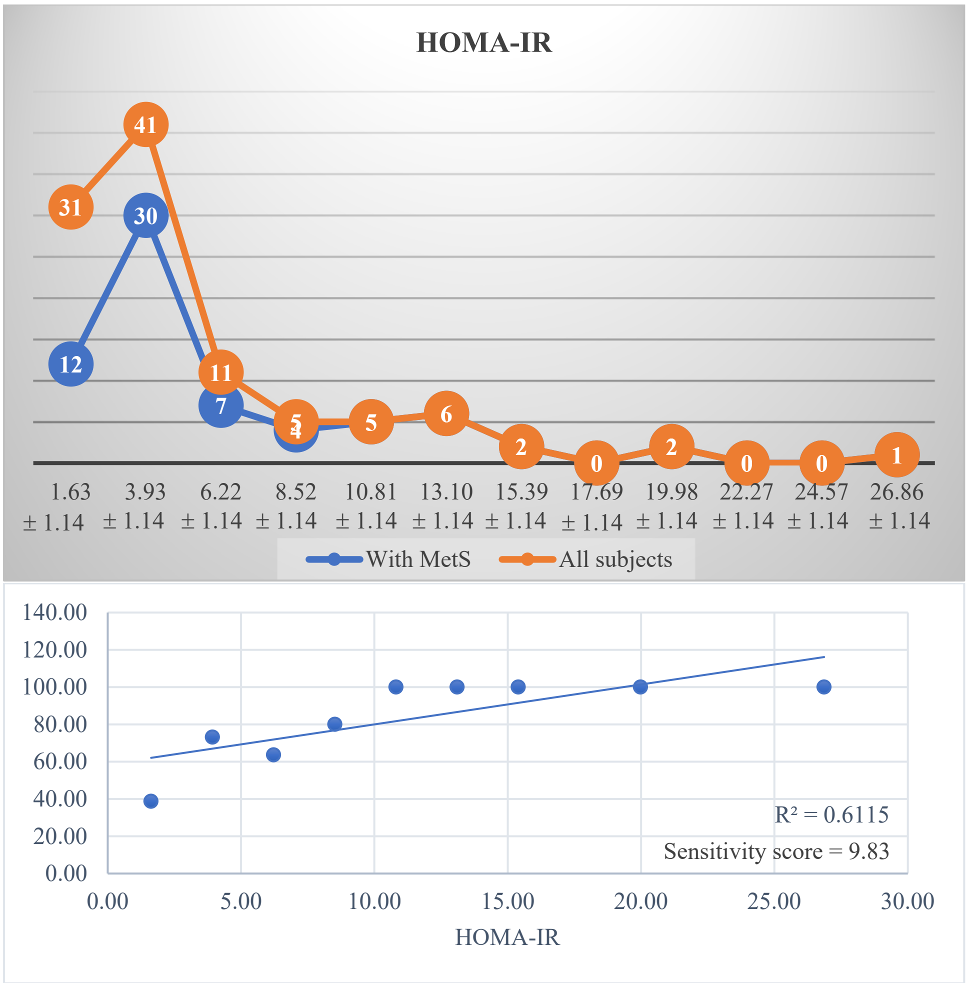

Figure S4. Distribution and prevalence charts for HOMA-IR

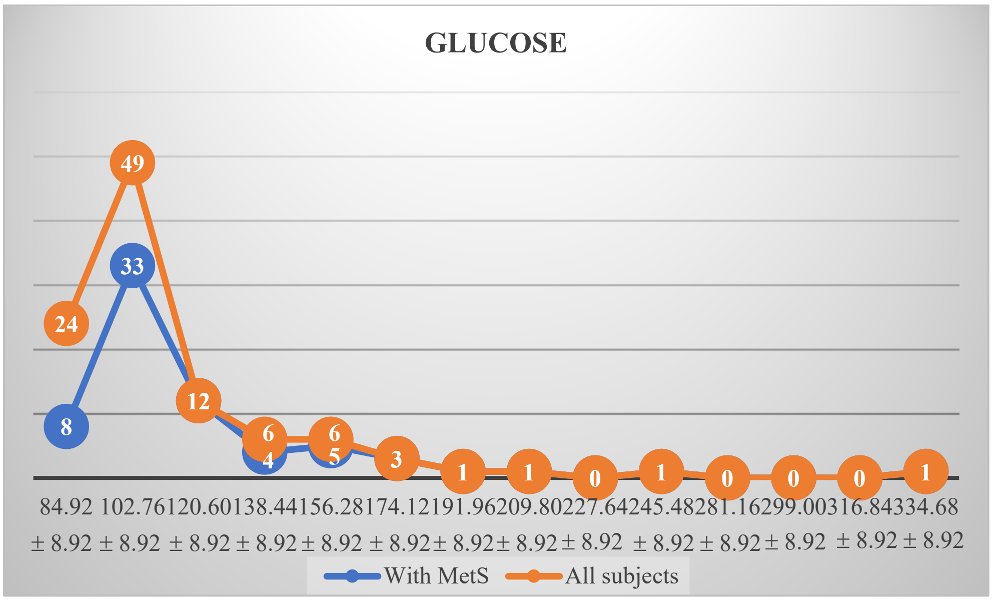

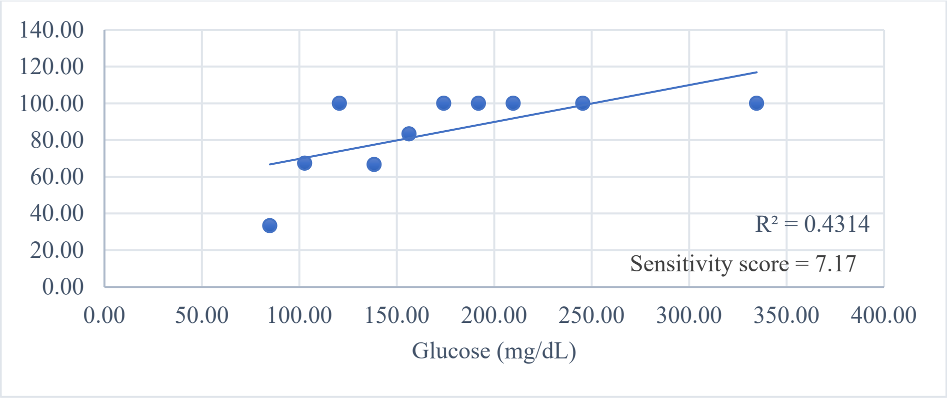

Figure S5. Distribution and prevalence charts for Glucose

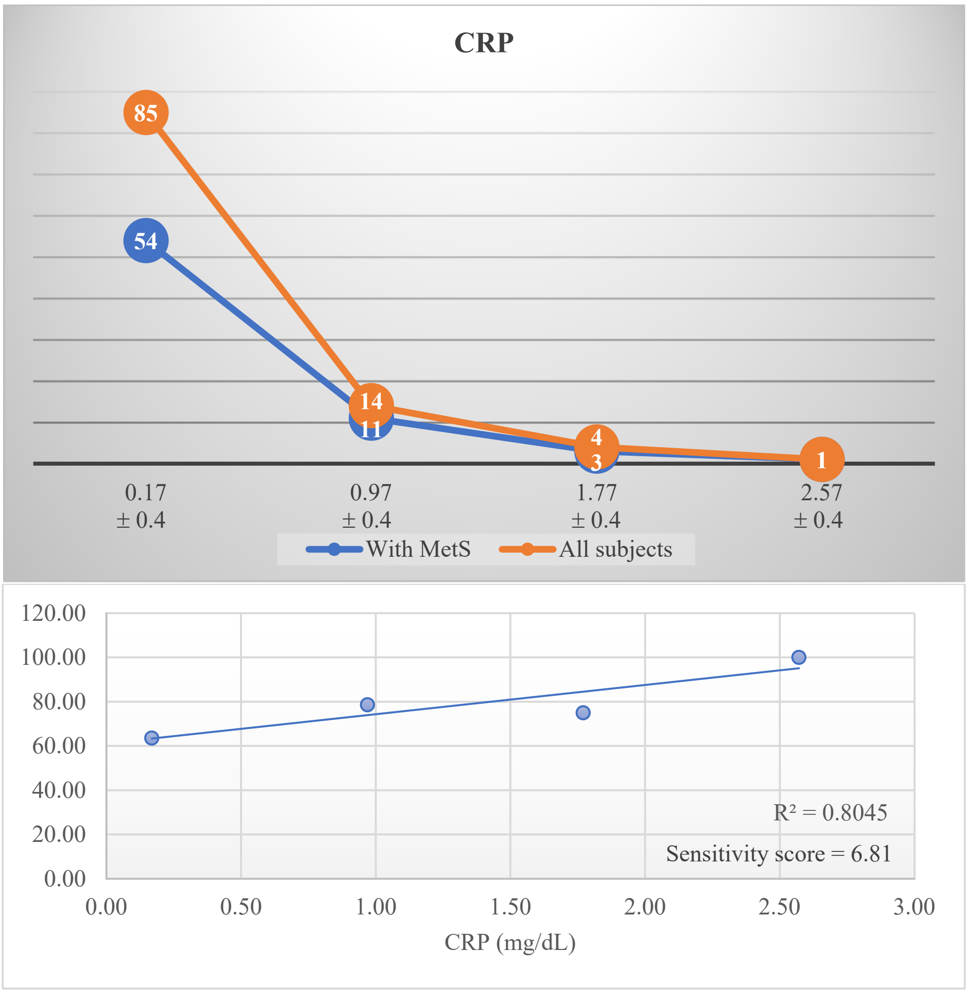

Figure S6. Distribution and prevalence charts for CRP

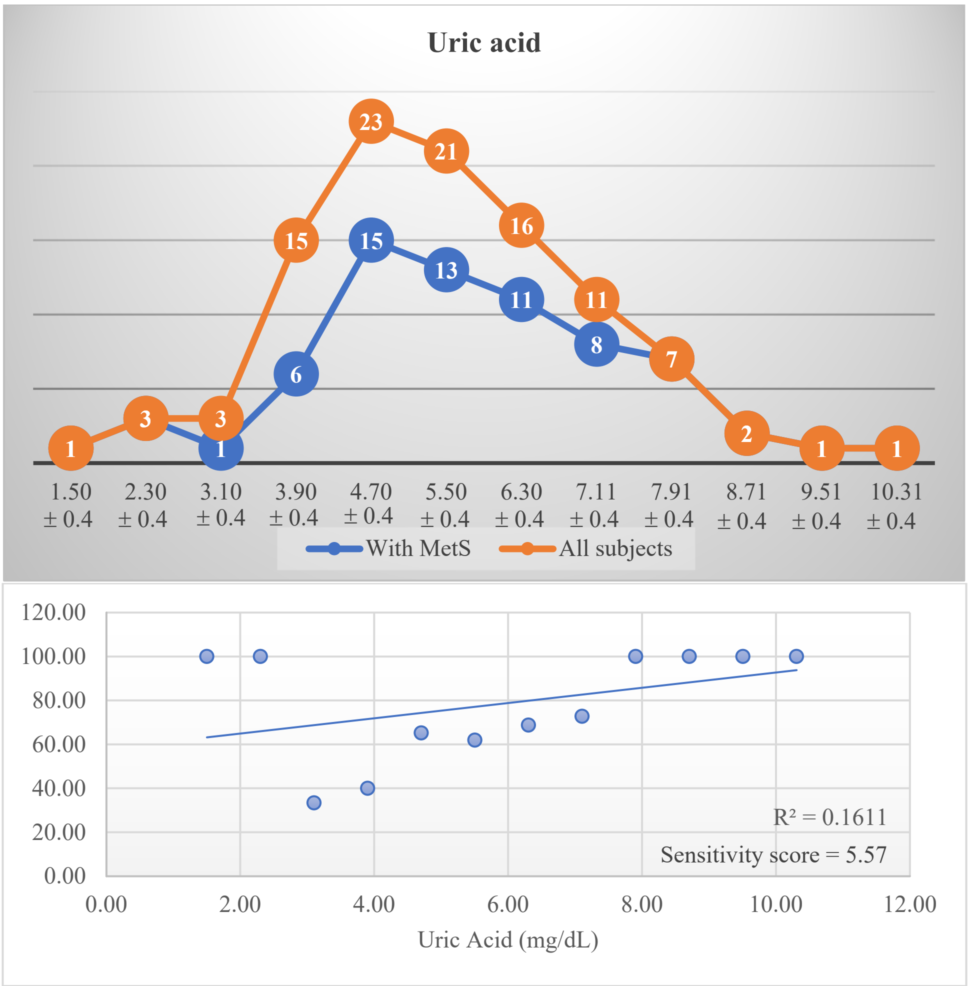

Figure S7. Distribution and prevalence charts for Uric acid

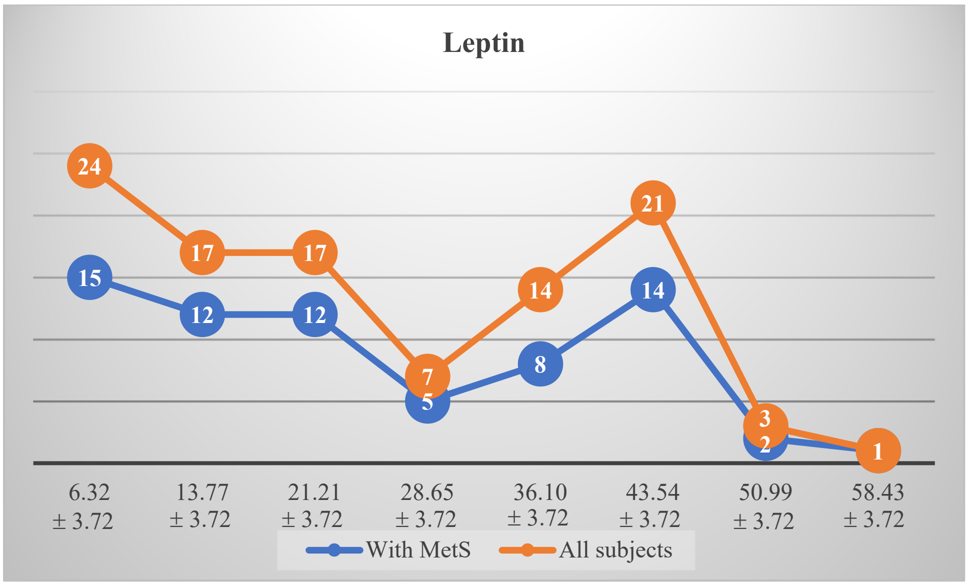

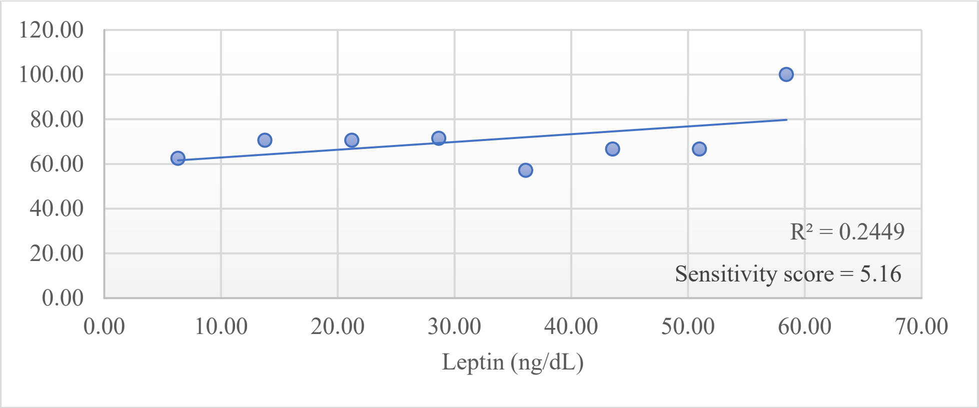

Figure S8. Distribution and prevalence charts for Leptin

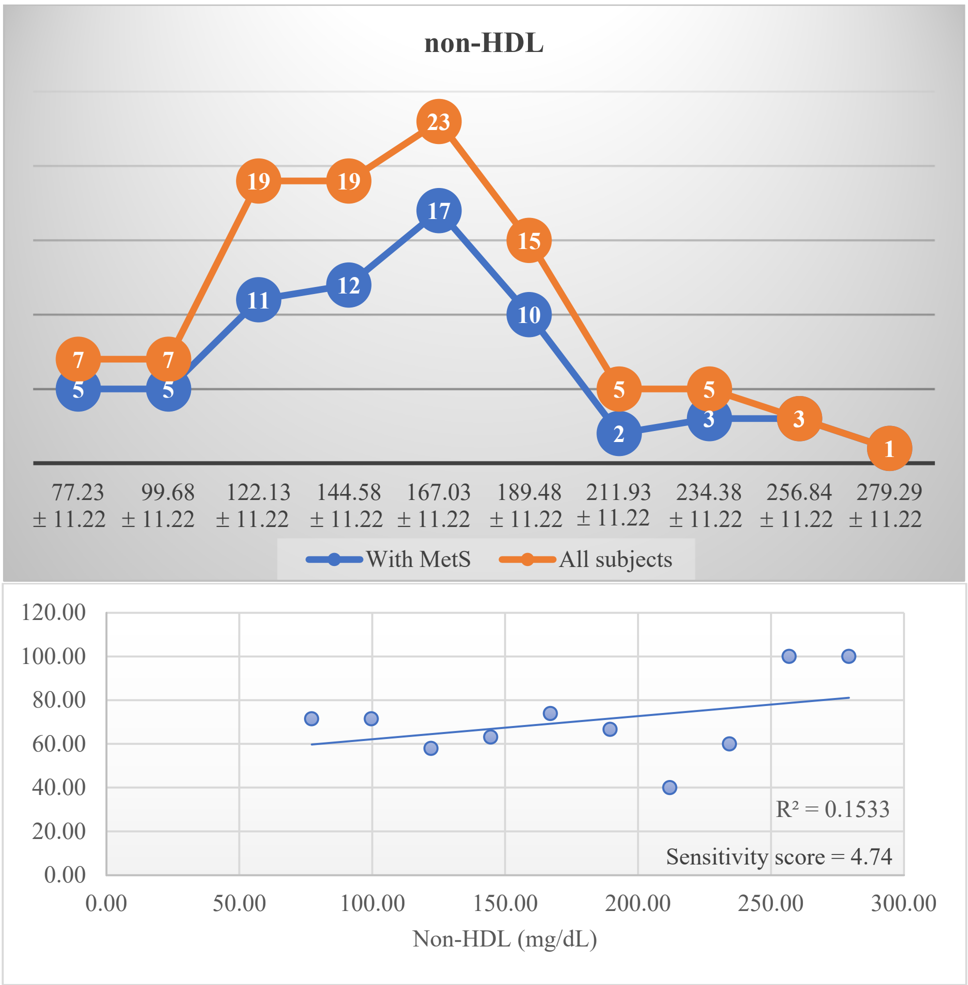

Figure S9. Distribution and prevalence charts for non-HDL

Distribution and prevalence charts for body composition parameters in descending order of their influence towards MetS

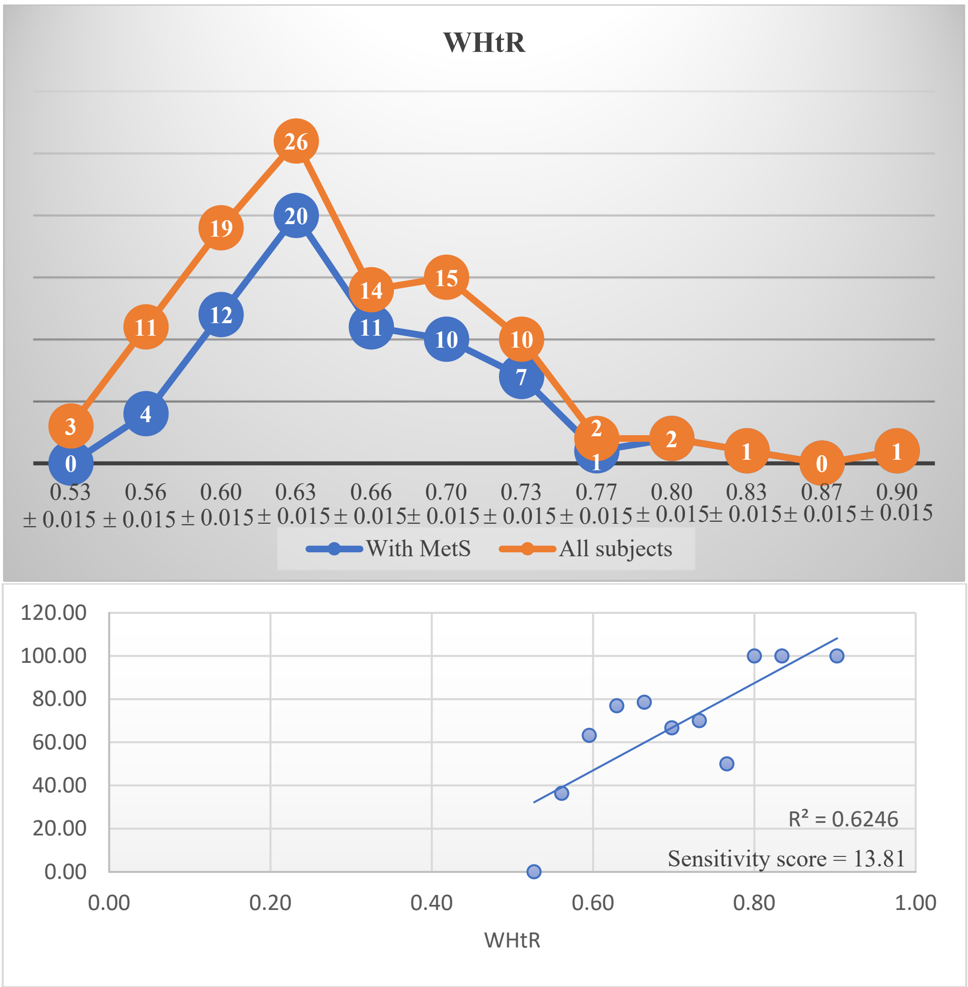

Figure S10. Distribution and prevalence charts for WHtR (waist-height ratio)

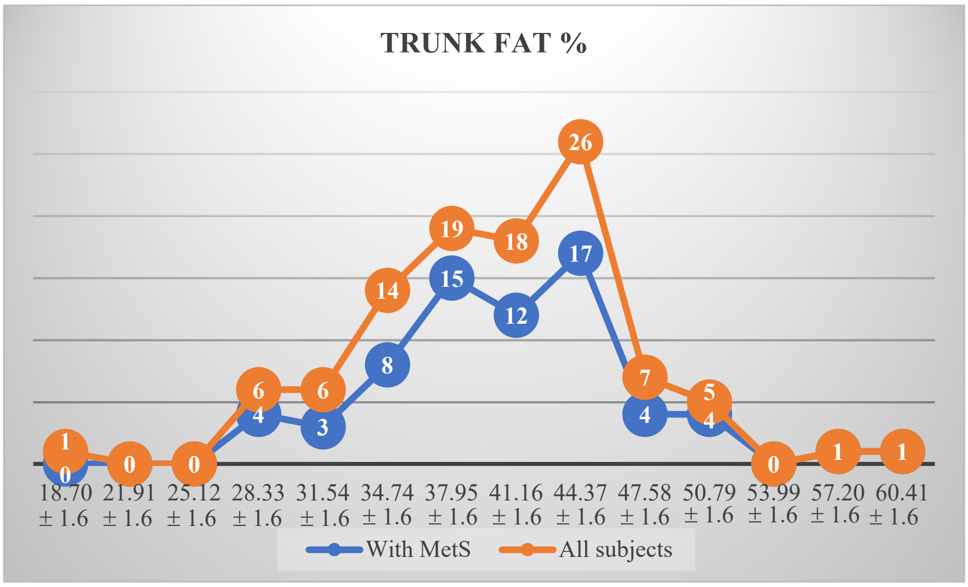

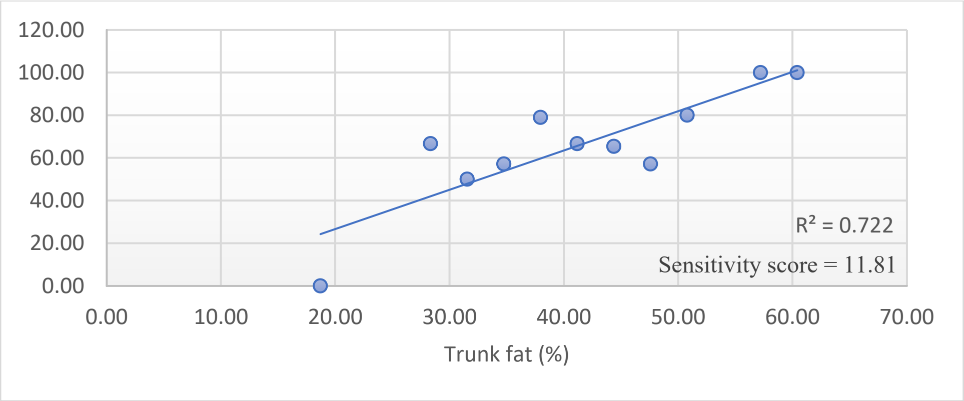

Figure S11. Distribution and prevalence charts for Trunk fat (%)

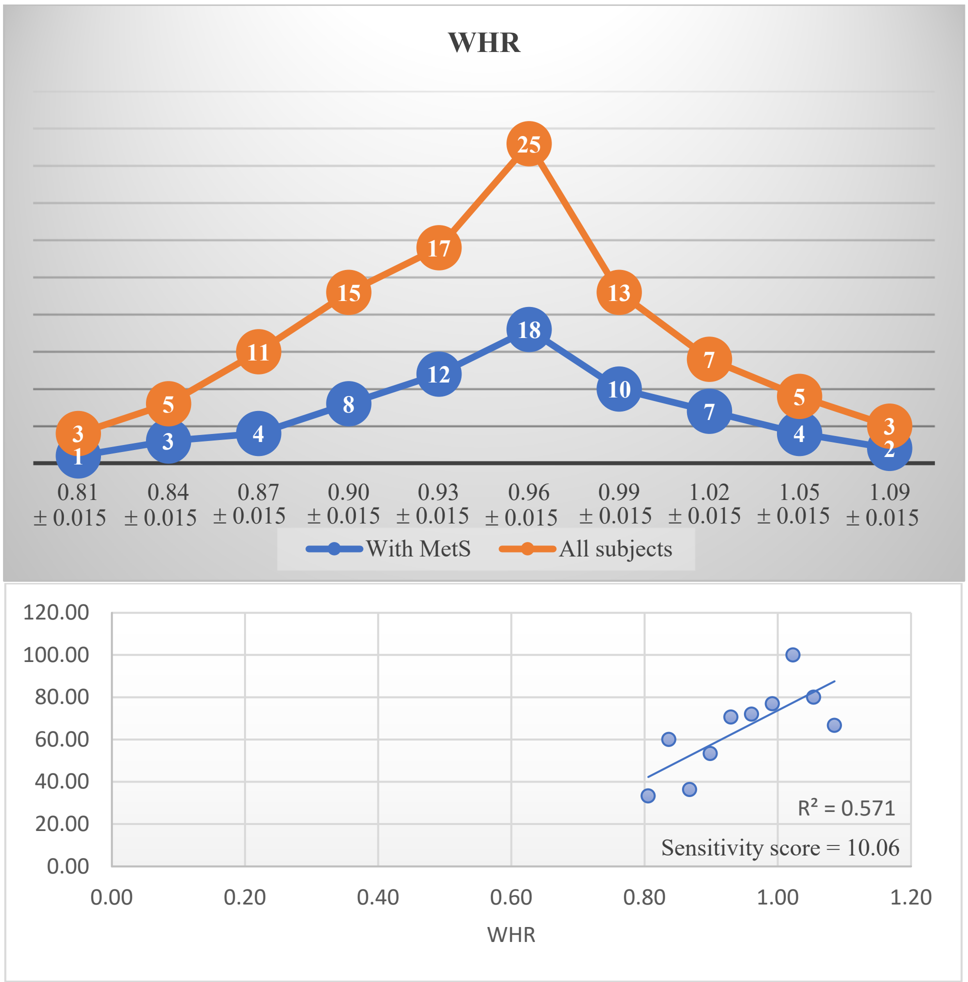

Figure S12. Distribution and prevalence charts for WHR (waist-hip ratio)

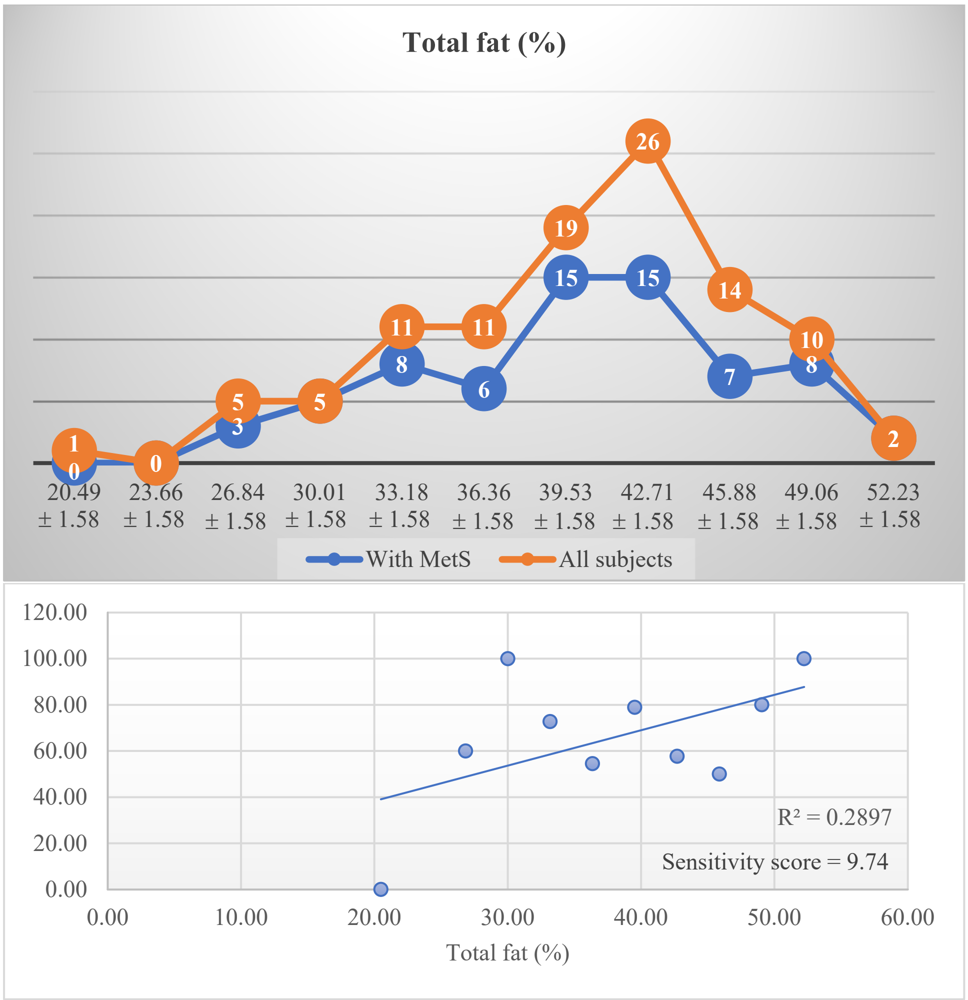

Figure S13. Distribution and prevalence charts for Total fat (%)

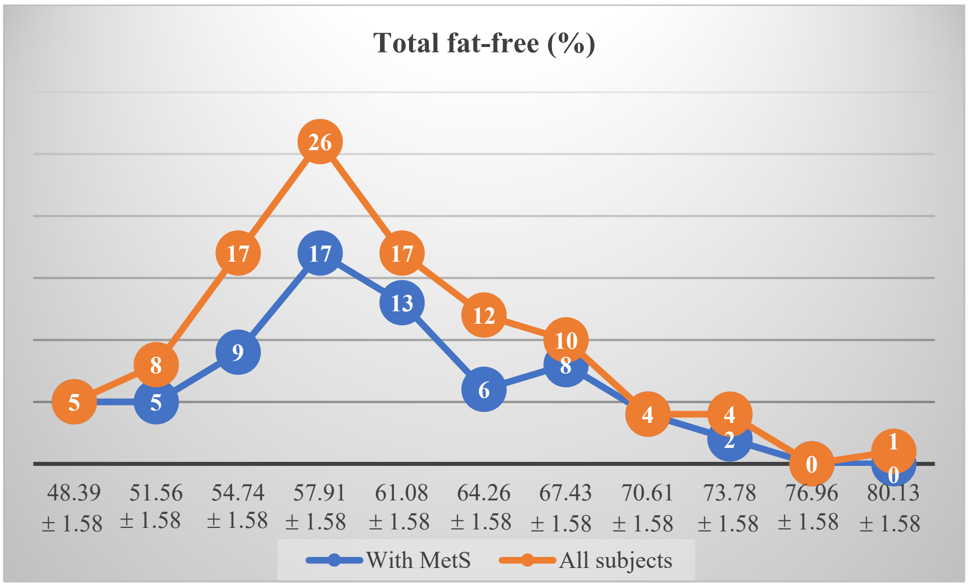

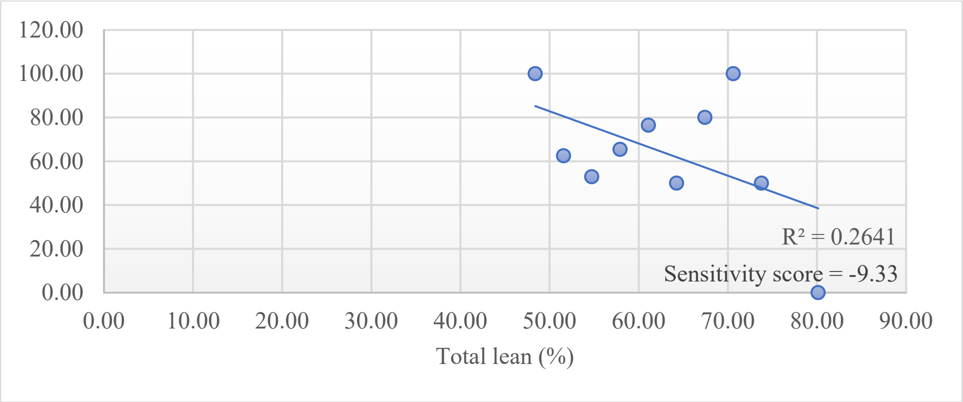

Figure S14. Distribution and prevalence charts for Total lean (%)

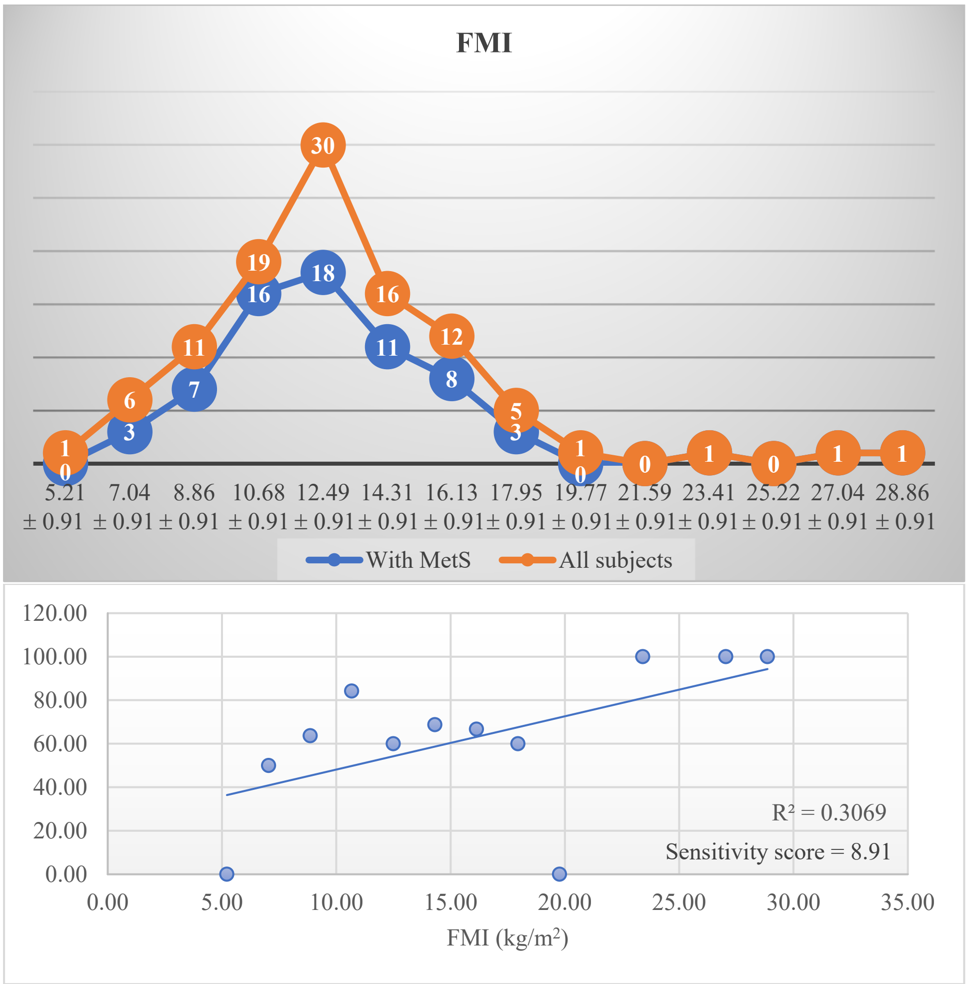

Figure S15. Distribution and prevalence charts for FMI (fat mass index)

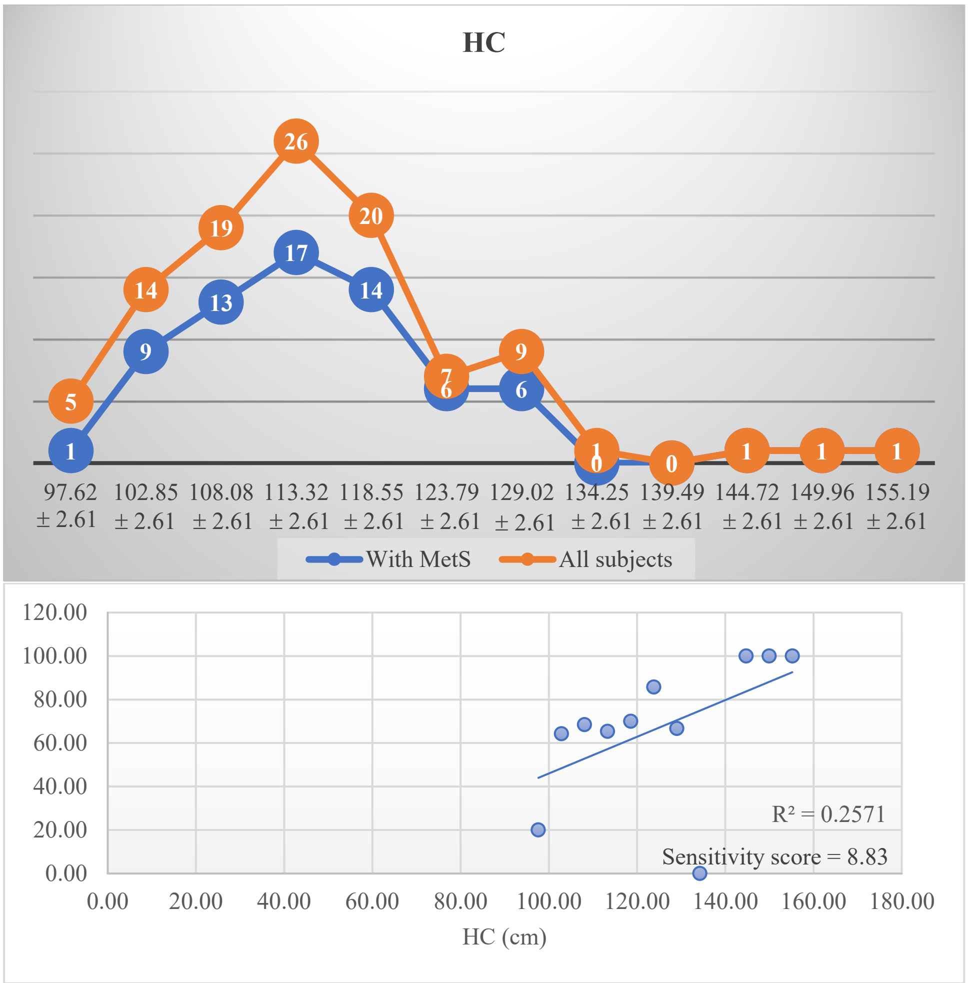

Figure S16. Distribution and prevalence charts for HC (hip circumference)

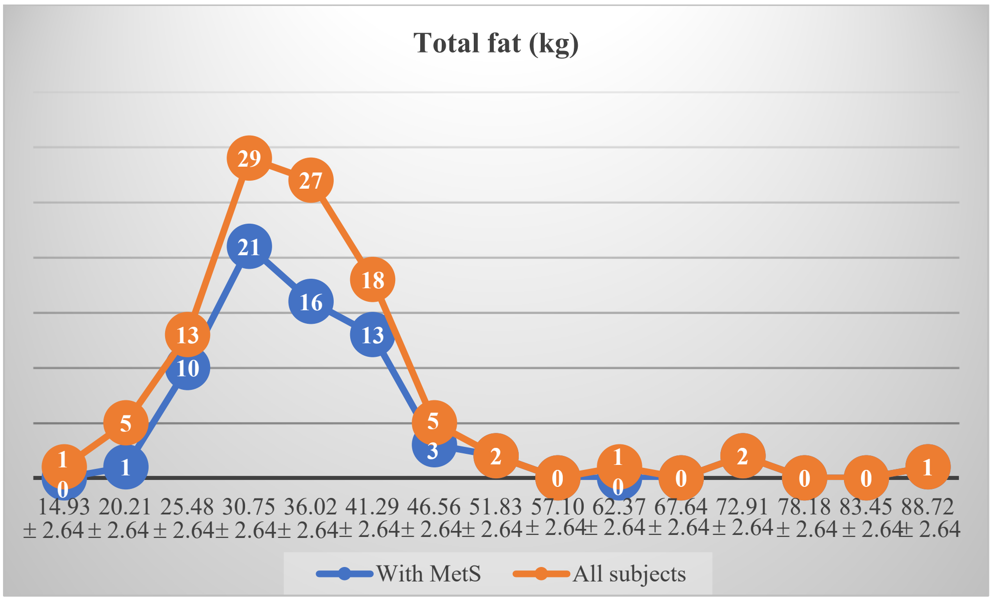

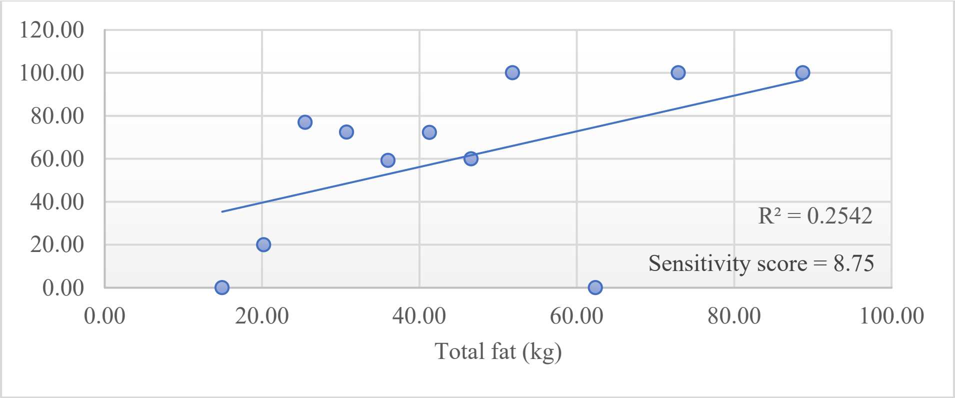

Figure S17. Distribution and prevalence charts for Total fat (kg)

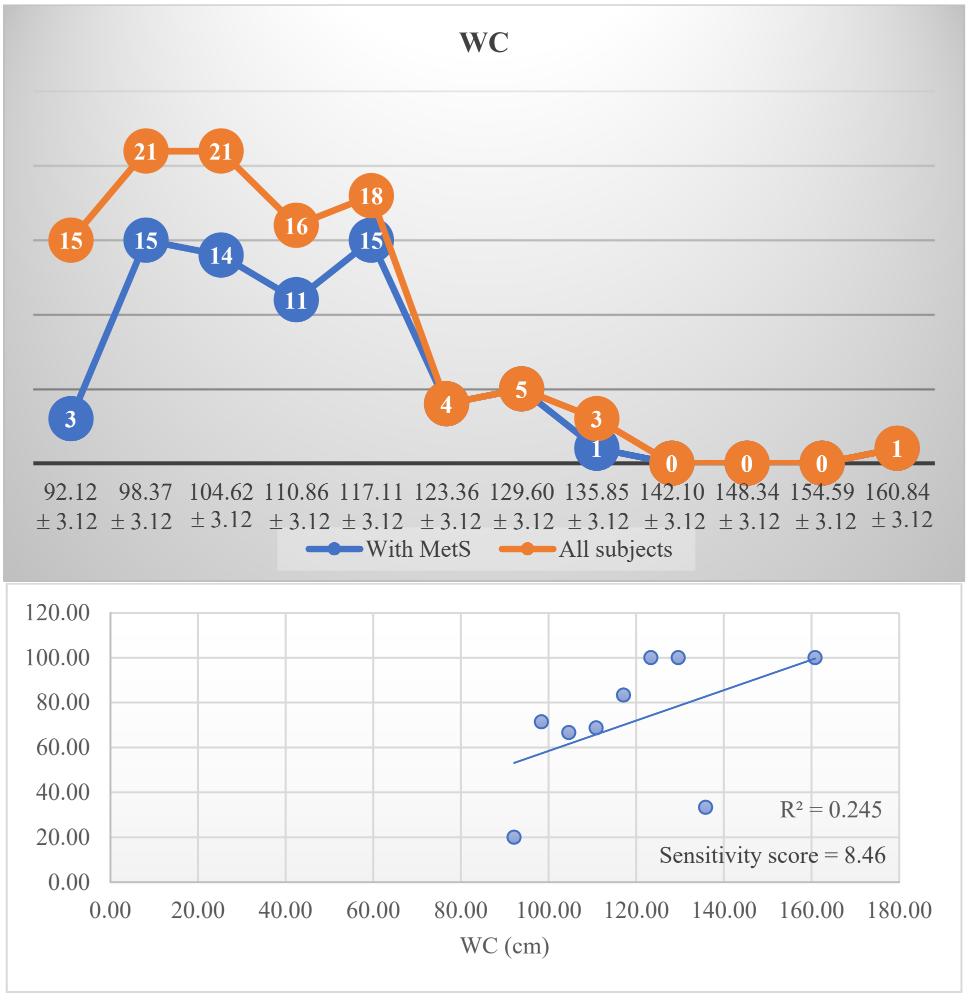

Figure S18. Distribution and prevalence charts for WC (waist circumference)

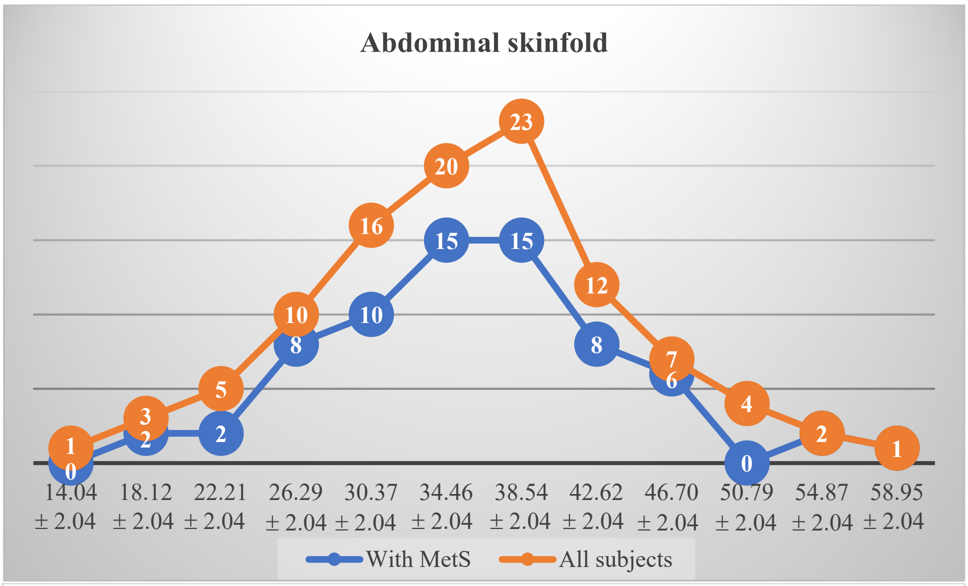

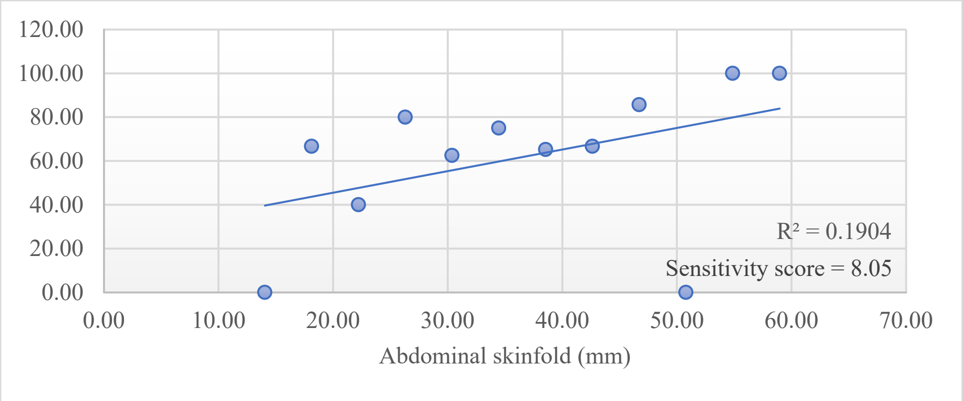

Figure S19. Distribution and prevalence charts for Abdominal skinfold

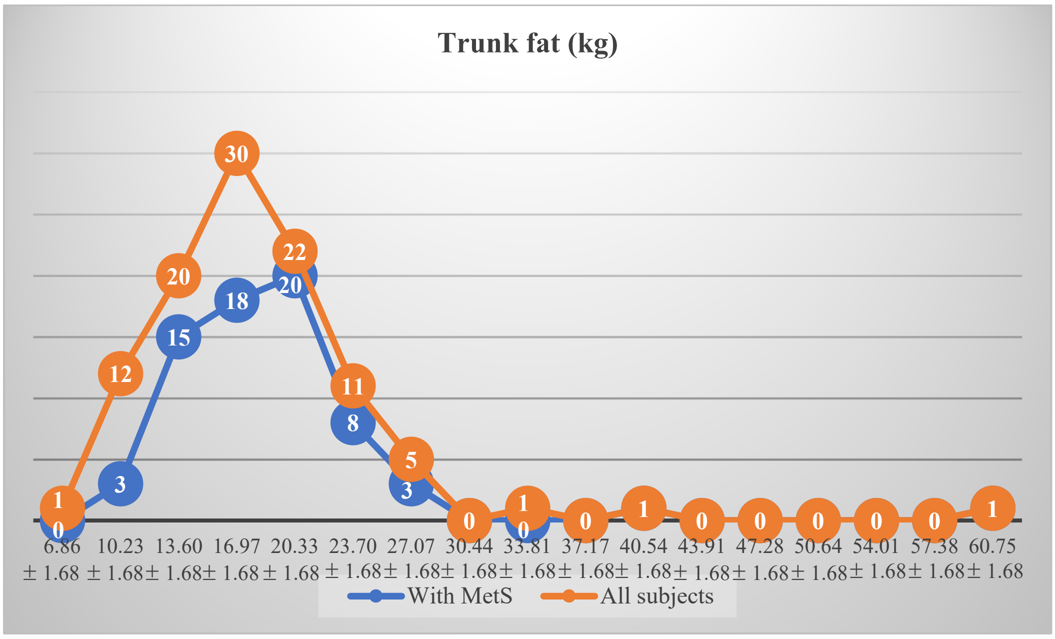

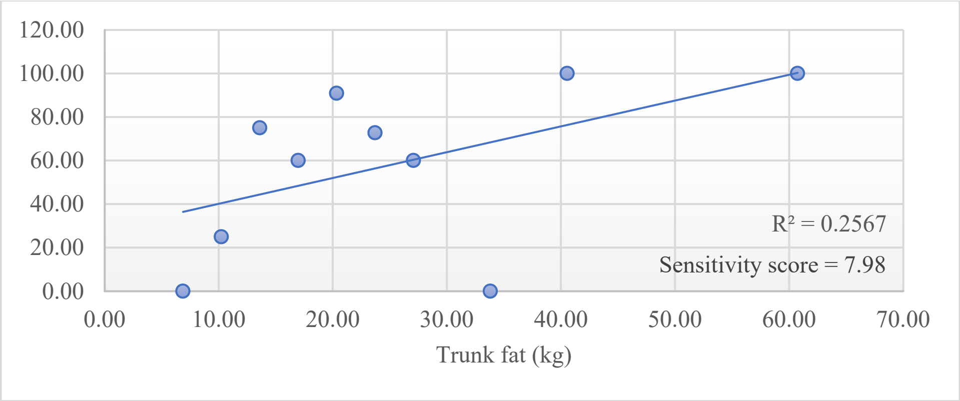

Figure S20. Distribution and prevalence charts for Trunk fat (kg)

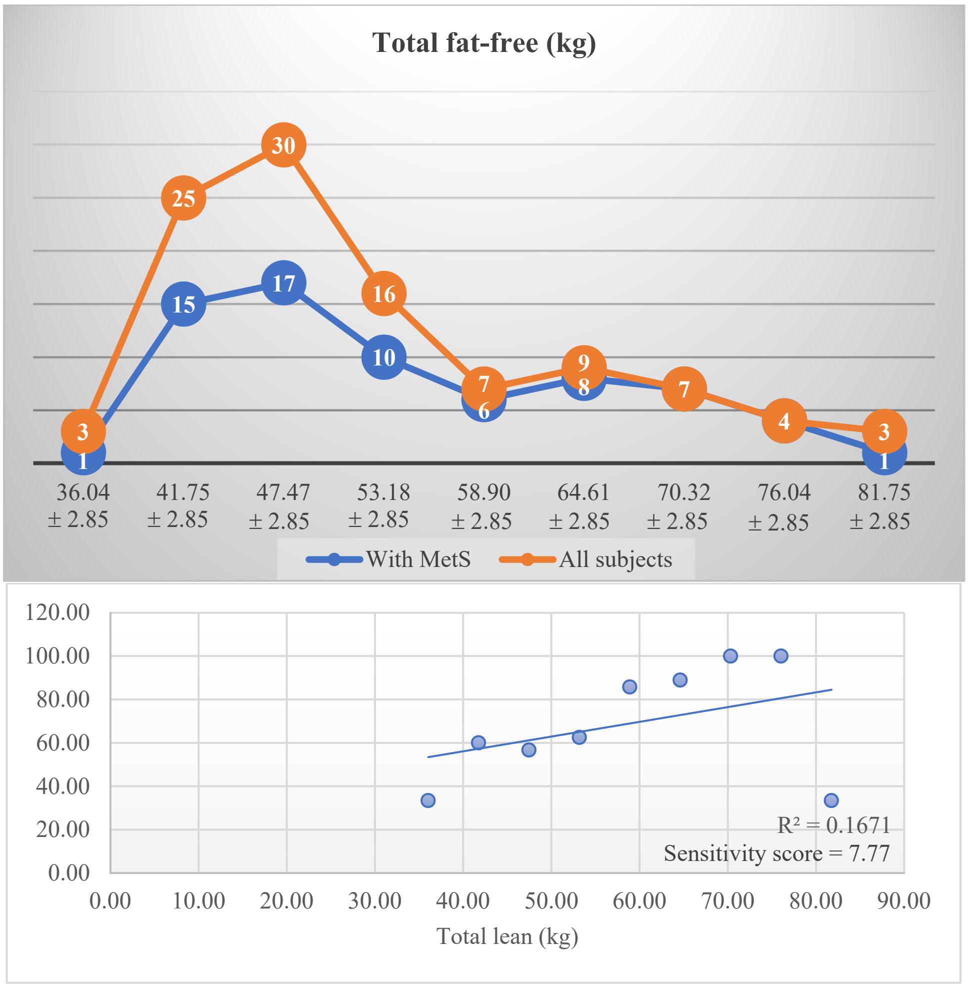

Figure S21. Distribution and prevalence charts for Total lean (kg)

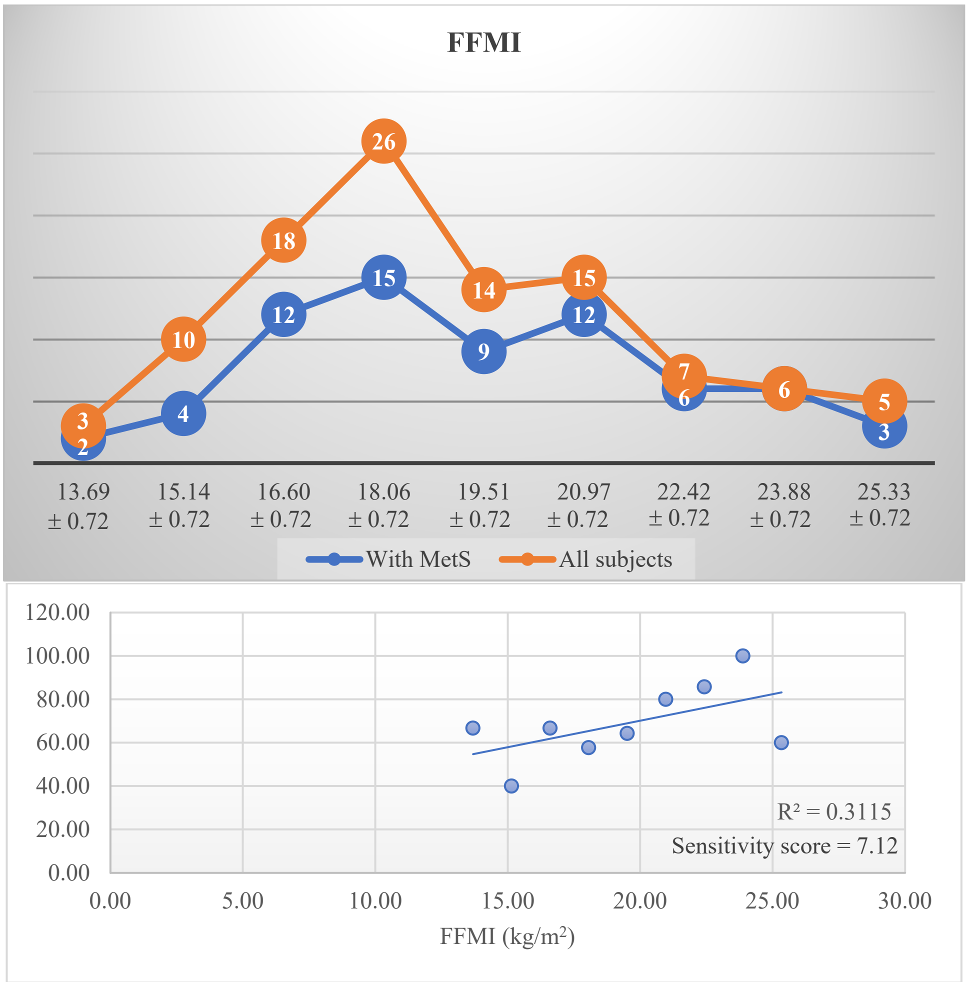

Figure S22. Distribution and prevalence charts for FFMI (fat free mass index)

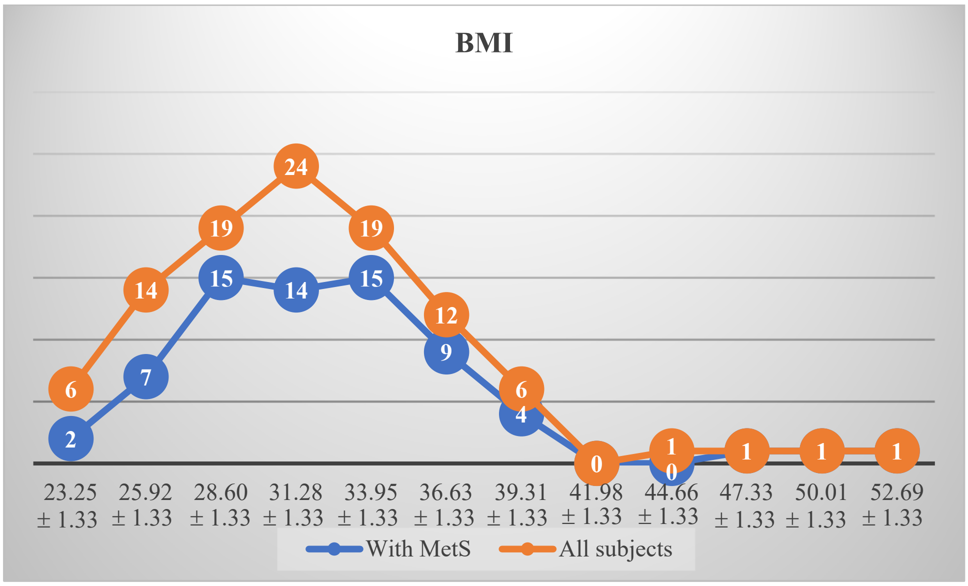

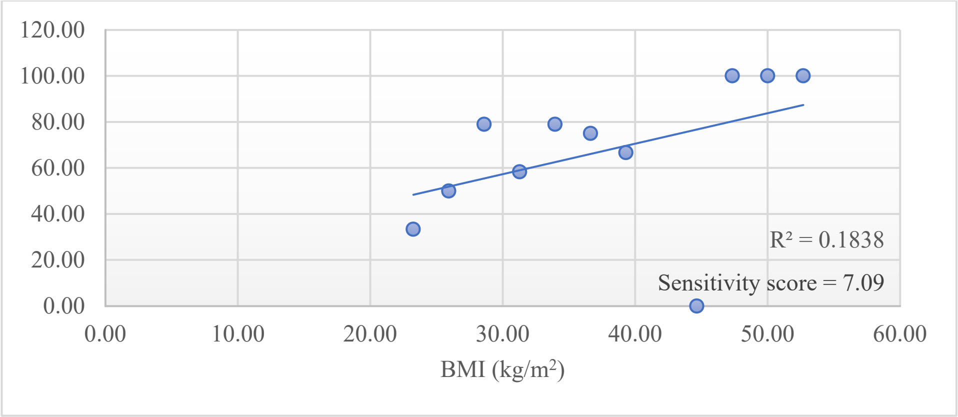

Figure S23. Distribution and prevalence charts for BMI

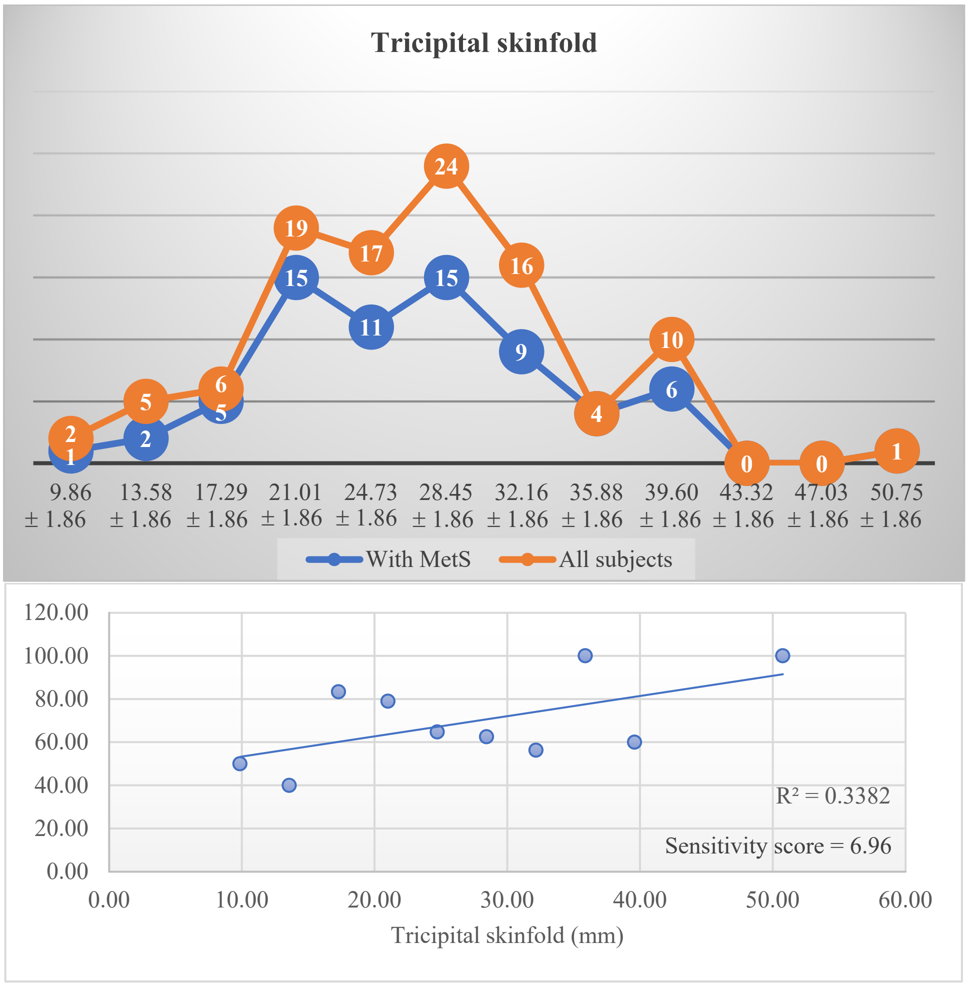

Figure S24. Distribution and prevalence charts for Tricipital skinfold
